# An Inflammatory Response-Related Gene Signature Can Impact the Immune Status and Predict the Prognosis of Hepatocellular Carcinoma

**DOI:** 10.3389/fonc.2021.644416

**Published:** 2021-03-22

**Authors:** Zhuo Lin, Qian Xu, Dan Miao, Fujun Yu

**Affiliations:** ^1^ Department of Hepatology, The First Affiliated Hospital of Wenzhou Medical University, Wenzhou, China; ^2^ Department of Accurate Diagnosis and Treatment of Chronic Liver Diseases, Key Laboratory of Zhejiang Province, Wenzhou, China; ^3^ Department of Gastroenterology, The First Affiliated Hospital of Wenzhou Medical University, Wenzhou, China

**Keywords:** hepatocellular carcinoma, inflammatory response, gene signature, overall survival, immune status, tumor microenvironment, drug sensitivity

## Abstract

**Background:**

Hepatocellular carcinoma (HCC) is a highly heterogeneous disease, which makes the prognostic prediction challenging. As part of the active cross-talk between the tumor and the host, inflammatory response in the tumor or its microenvironment could affect prognosis. However, the prognostic value of inflammatory response-related genes in HCC remains to be further elucidated.

**Methods:**

In this study, the mRNA expression profiles and corresponding clinical data of HCC patients were downloaded from the public database. The least absolute shrinkage and selection operator Cox analysis was utilized to construct a multigene prognostic signature in the TCGA cohort. HCC patients from the ICGC cohort were used for validation. Kaplan Meier analysis was used to compare the overall survival (OS) between high- and low-risk groups. Univariate and multivariate Cox analyses were applied to determine the independent predictors for OS. Single-sample gene set enrichment analysis was utilized to calculate the immune cell infiltration score and immune related pathway activity. Gene set enrichment analysis was implemented to conduct GO terms and KEGG pathways. The qRT-PCR and immunohistochemistry were utilized to perform the mRNA and protein expression of prognostic genes between HCC tissues and normal liver tissues respectively.

**Results:**

An inflammatory response-related gene signature model was constructed by LASSO Cox regression analysis. Compared with the low-risk group, patients in the high-risk group showed significantly reduced OS. Receiver operating characteristic curve analysis confirmed the predictive capacity of the prognostic gene signature. Multivariate Cox analysis revealed that the risk score was an independent predictor for OS. Functional analysis indicated that immune status was definitely different between two risk groups, and cancer-related pathways were enriched in high-risk group. The risk score was significantly correlated with tumor grade, tumor stage and immune infiltrate types. The expression levels of prognostic genes were significantly correlated with sensitivity of cancer cells to anti-tumor drugs. Furthermore, the expression of prognostic genes showed significant difference between HCC tissues and adjacent non-tumorous tissues in the separate sample cohort.

**Conclusion:**

A novel signature constructed with eight inflammatory response-related genes can be used for prognostic prediction and impact the immune status in HCC. Moreover, inhibition of these genes may be a therapeutic alternative.

## Introduction

Liver cancer is the sixth most commonly diagnosed cancer and the fourth leading cause of cancer death worldwide (1). Hepatocellular carcinoma (HCC) accounts for the majority of primary liver cancer. Etiologies for HCC include chronic infection with hepatitis B virus and hepatitis C virus, alcohol addiction, metabolic liver disease (especially nonalcoholic fatty liver disease) and exposure to dietary toxins such as aflatoxin and aristolochic acid (2). HCC is a complex and heterogeneous disease with a 5-year survival rate of only 14.1% in China due to the high frequency of recurrence (3), usually accompanied by cirrhosis or other related complications that bring great challenges to the prognosis evaluation.

The link between inflammation and cancer is well recognized. Rudolf Welshaw et al. first discovered “lymphatic network infiltration” near the origin of cancer, and pointed out that it plays an active role in the occurrence of cancer (4). The role of inflammation in the occurrence and development of cancer has always been the focus of people’s research (4–7). Inflammation can both promote and inhibit cancers (5, 8). By analyzing the routinely available parameters in the blood, people can explore the relationship of cancer with inflammatory markers. For example, studies confirmed many inflammatory response-related features in the peripheral blood of patients with liver cancer, such as thrombocytosis, leukocytosis, hypoproteinemia and elevated plasma fibrinogen (9). The clinical systemic inflammation markers including medium-granulocyte ratio, platelet-lymphoid ratio and lymphoid-monocyte ratio were evaluated in newly diagnosed and previously untreated HCC, and these markers showed significant prognostic ability for OS independent of previously recognized prognostic factors for HCC (10). The Glasgow prognosis score composed of C-reactive protein and albumin had independent prognostic value for cancer patients (11). More and more studies supported the combination of various acute phase proteins to develop comprehensive prognostic scores for cancers based on inflammation. In addition to serum markers, some inflammatory response-related genes were used to predict the metastatic potential of HCC (12). However, the relationship between inflammatory response-related genes and the prognosis of HCC remains unknown.

In this study, we downloaded the mRNA expression profile and corresponding clinical data of patients with HCC from the public database. Then, we constructed a prognostic signature with differentially expressed genes (DEGs) related to inflammatory response in the TCGA cohort and validated the stability and reliability of the model through the ICGC cohort. Then, we further carried out functional enrichment analysis to explore its potential mechanism. Besides, we analyzed the association between prognostic gene expression and immune infiltrate types. Moreover, we investigated the relationship of prognostic gene expression with tumor stemness and cancer chemoresistance. Finally, the mRNA and protein expression of prognostic genes between HCC tissues and adjacent non-tumorous tissues was validated by laboratory experiments.

## Methods

### Data Collection (TCGA-LIHC Cohort and ICGC (LIRI-JP) Cohort)

RNA sequencing data and corresponding clinical information of 370 patients with liver cancer were downloaded from TCGA website (https://portal.gdc.cancer.gov/repository). RNA sequencing data and clinical information of another 231 tumor samples were obtained from ICGA website (https://dcc.icgc.org/projects/LIRI-JP). These samples were mainly derived from Japanese people infected with hepatitis B virus or hepatitis C virus. The data from TCGA and ICGC were both public, following the data access policy and publication guidelines of TCGA and ICGC. Then, 200 inflammatory response-related genes were found in the Molecular Signatures database and provided in the **Supplementary Table 1**.

### Construction and Validation of a Prognostic Inflammatory Response-Related Gene Signature

DEGs between tumor tissues and non-tumor tissues were identified by “limma” R package with fold change > 2 and a false discovery rate < 0.05 in TCGA cohort. Univariate Cox analysis was used to screen the inflammatory response-related genes with prognostic value, and the P value was adjusted by Benjamini & Hochberg (BH) correction method. LASSO-penalized Cox regression analysis was utilized to construct a prognostic model in order to minimize the risk of overfitting (13, 14). The LASSO algorithm was used to select and shrink variables with “glmnet” R package, so that some regression coefficients were strictly equal to 0, thereby obtaining an interpretable model. The normalized expression matrix of candidate prognostic DEGs was the independent variable in regression, and the dependent variable was the overall survival and status of patients in the TCGA cohort. The tenfold cross-validation was used to determine the penalty parameter (λ) of the prognostic model and was followed the minimum criteria (i.e. the value of λ corresponding to the lowest partial likelihood deviance). The risk scores of patients were calculated according to the expression level of each inflammatory response-related gene and its corresponding regression coefficient. The formula was established as follows: score= e^sum (each gene’s expression × corresponding coefficient)^. According to the median risk score, patients were divided into high- and low-risk groups. In terms of expression levels of genes in the constructed model, PCA analysis and t-SNE analysis were performed with “Rtsne” and “ggplot2” R packages to explore the distribution of different groups. The survival analysis was implemented to analyze the OS of high- and low-risk groups using the “survminer” R package. The “survival” R package and “timeROC” R package were carried out to conduct time‐dependent ROC curve analysis in order to evaluate the predictive value of the prognostic signature. Furthermore, univariate and multivariate Cox analyses were performed to explore the independent prognostic value of the 8-gene signature.

### Functional Enrichment Analysis

Gene set enrichment analysis (GSEA) was utilized to conduct Gene Ontology (GO) and Kyoto Encyclopedia of Genes and Genomes (KEGG) analyses with GSEA software 4.1 based on the DEGs between the high- and low-risk groups. P value was adjusted by BH method. The infiltration scores of 16 immune cells and the activities of 13 immune-related pathways between the high- and low-risk groups were calculated by single-sample gene set enrichment analysis (ssGSEA) with the “GSVA” R package.

### Tumor Microenvironment and Immune Response Analysis

The infiltration levels of immune cells and stromal cells in different tumor tissues were analyzed by immune score and stromal score (15). Spearman correlation was utilized to test the correlation between risk score and those scores. The association between risk score and immune infiltration subtype was tested by 2-way ANOVA analysis. Tumor stem cell features extracted from transcriptome and epigenetics of TCGA tumor samples were used to measure stem cell-like features of tumor (16). The association of tumor stemness with risk score was analyzed by Spearman correlation test.

### Chemotherapy Sensitivity Analysis

The NCI-60 database containing 60 different cancer cell lines from 9 different types of tumors was accessed through the CellMiner interface (https://discover.nci.nih.gov/cellminer). Pearson correlation analysis was performed to investigate the association between the prognostic gene expression and drug sensitivity. Correlation analysis was made on the efficacy of 263 drugs approved by FDA or in clinical trials (**Supplementary Table 2**).

### Verification of the mRNA Expression of Prognostic Genes Between HCC Tissues and Adjacent Non-Tumorous Tissues by qRT-PCR

A total of twenty paired HCC and adjacent non-tumorous tissue samples were collected from the First Affiliated Hospital of Wenzhou Medical University. Ethics approval was granted by Human Research Ethics Committee in The First Affiliated Hospital of Wenzhou Medical University. The mRNA expression levels of eight prognostic genes in samples were detected by qRT-PCR method. According to the manufacturer’s instruction, the total RNA of HCC and adjacent normal liver tissue samples was prepared with Trizol reagent (Servicebio). Then, RNA was reverse-transcribed into cDNA using RevertAid First Strand cDNA Synthesis Kit (Thermo). Gene expression was standardized as GAPDH. FastStart Universal SYBR Green Master (Roche) was utilized to quantify the real-time PCR analysis by StepOne (Applied Biosystems). The sequence of primers was shown in **Supplementary Table 3**. Each RNA sample was performed in triplicate. In order to compare the expression levels of different samples, the relative expression of inflammatory response-related genes was calculated by 2^−ΔΔCt^ method.

### Verification of the Protein Expression of Prognostic Genes Between HCC Tissues and Adjacent Non-Tumorous Tissues by Immunohistochemistry (IHC)

A total of ten paired HCC and adjacent non-tumorous tissue samples were collected from the First Affiliated Hospital of Wenzhou Medical University with permission from the ethics committees of The First Affiliated Hospital of Wenzhou Medical University. The expression levels of eight prognostic genes in ten pairs of HCC and adjacent non-tumorous tissues were validated by IHC experiment. All specimens were fixed with 10% formalin at room temperature, embedded in paraffin and processed into 4 μm serial sections. Briefly, the tissue slices were dewaxed, then hydrated and boiled in 10 mmol/L citrate buffer (pH=6.4) for 10 minutes to recover the antigen. After that, the slices were treated with methanol containing 3% hydrogen peroxide to inactivate the endogenous peroxidase and treated with citric acid buffer (pH=6.0) to obtain optimal antigen recovery. The 1% bovine serum albumin was incubated in phosphate buffer for 30 minutes to block non-specific binding. In addition, the slices were stained with primary antibody and incubated overnight at 4°C. Then, these sections were treated with three 5-min mild washing in phosphate buffer saline, followed by staining with secondary antibody (HRP polymer) at 1:200 for 50 minutes. Diaminobenzidine was applied before being counterstained with hematoxylin. Finally, the samples were sealed, observed and photographed by light microscope. The primary antibodies used in our work were as follows: Anti-ADORA2B antibody (1:200, ER1903-44, HUABIO), Anti-Integrin alpha 5 antibody [JJ08-94] (1:50, ET1701-58, HUABIO), Rabbit Anti-MEP1A/Meprin alpha antibody (1:100, bs-6056R, BIOSS), NOD2 Antibody - N-terminal (1:100, DF12125, Affinity), P2RX4 Polyclonal Antibody (1:100, 13534-1-AP, Proteintech), Anti-RIP2 antibody (1:200, ER1915-87, HUABIO), Anti-SERPINE1 antibody [H9-D5] (1:150, EM1709-36, HUABIO), CAT-1 Polyclonal Antibody (1:100, 14195-1-AP, Proteintech). Primary antibody information was summarized in **Supplementary Table 5**.

### Statistical Analysis

DEGs between tumor tissues and adjacent tissues were compared by WilCoxon test. The Chi-squared test was used to compare the different proportions. The ssGSEA scores of immune cells or immune pathways between high- and low-risk groups were compared by Mann-Whitney test, and the P value was adjusted by BH method. Kaplan-Meier analysis was employed to compare the differences of OS among different groups. Univariate and multivariate Cox analyses were performed to screen the independent predictors for OS. The correlation of prognostic model risk score or prognostic gene expression level with stemness score, stromal score, immune score and drug sensitivity was tested by Spearman or Pearson correlation analysis. R software (Version 3.6.3) with packages venn, igraph, ggplot2, pheatmap, ggpubr, corrplot and survminer was used to create plots. In all statistical results, a two-tailed P value less than 0.05 indicated statistical significance.

## Results

**Figure 1:**
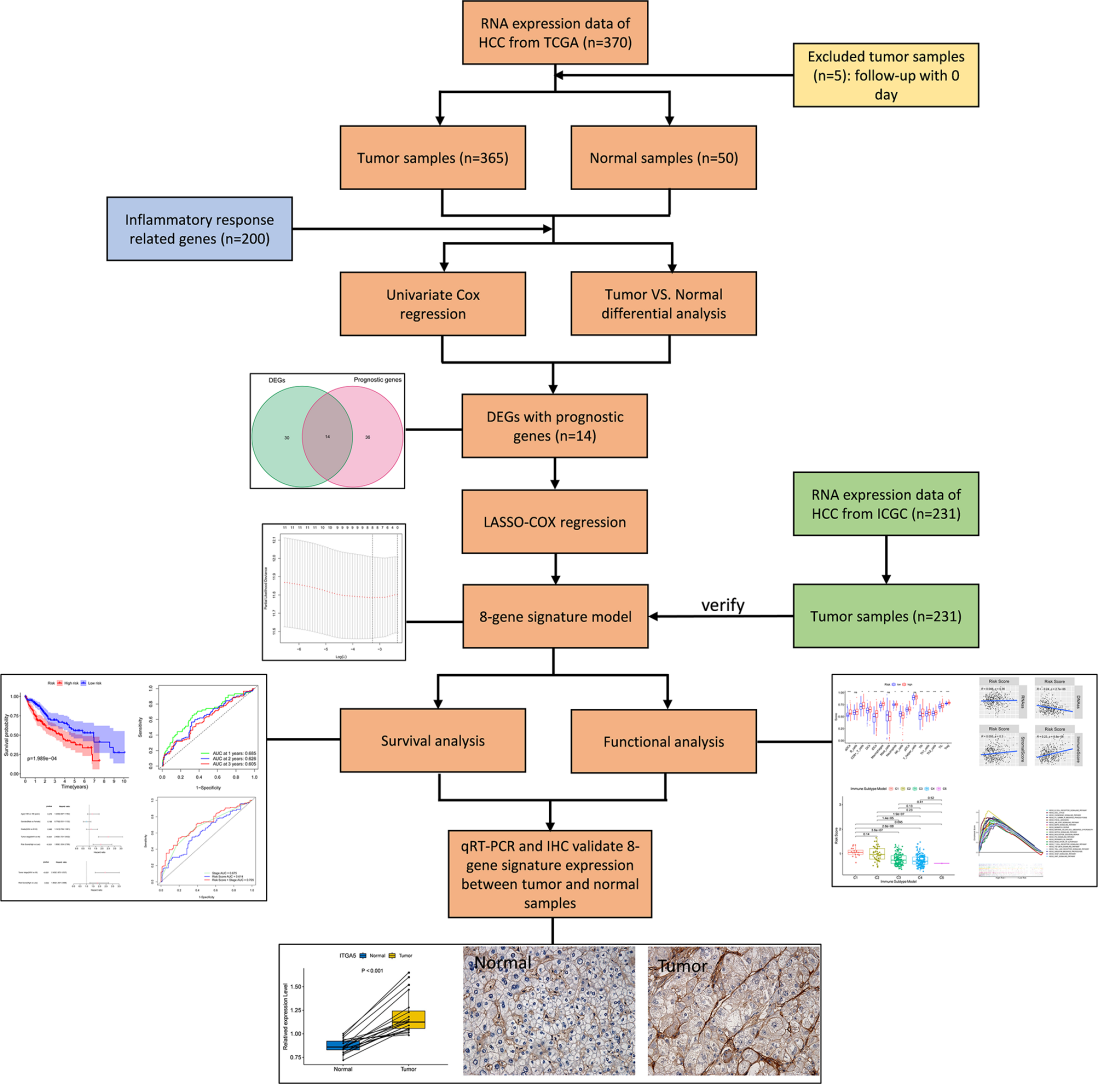
Flow chart of data collection, analysis and experiment.

The flow chart of this study was shown in **Figure 1**. The study population consisted of 365 HCC patients from TCGA-LIHC cohort and 231 HCC patients from ICGC (LIRI-JP) cohort. **Table 1** summarized the detailed clinical features of these patients.

**Table 1:** Clinical characteristics of the HCC patients used in this study.

	TCGA-LIHC cohort	ICGC-LIRP-JI cohort
**No. of patients**	365	231
**Age (median, range)**	57 (16–90)	67 (31–89)
**Gender**		
Female	119 (32.6%)	61 (26.4%)
Male	246 (67.4%)	170 (73.6%)
**Grade**		
Grade 1	55 (15.1%)	NA
Grade 2	175 (47.9%)	NA
Grade 3	118 (32.3%)	NA
Grade 4	12 (3.3%)	NA
Unknown	5 (1.4%)	NA
**Stage**		
I	170 (46.6%)	36 (15.6%)
II	84 (23.0%)	105 (45.5%)
III	83 (22.7%)	71 (30.7%)
IV	4 (1.1%)	19 (8.2%)
Unknown	24 (6.6%)	0 (0%)
**Survival status**		
Alive	235 (64.4%)	189 (81.8%)
Deceased	130 (35.6%)	42 (18.2%)

### Identification of Prognostic Inflammation-Related DEGs in the TCGA Cohort

**Figure 2:**
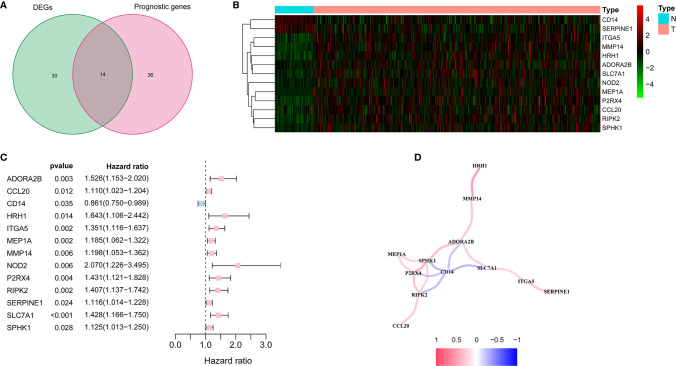
Identification of the candidate inflammatory response-related genes in the TCGA cohort. **(A)** Venn diagram to identify DEGs between HCC tissues and adjacent normal tissues. **(B)** The 13 overlapping genes expression between HCC tissues and adjacent normal tissues. **(C)** Forest plots showing the results of the association between 13 overlapping gene expression and OS. **(D)** The correlation network of candidate genes.

There were 44 inflammatory response-related genes differentially expressed in tumor tissues and adjacent non-tumorous tissues. Univariate Cox analysis showed that 14 of them were correlated with OS (**Figure 2A**). TACR3 was excluded from this analysis because its expression was 0 in more than 350 samples. The 13 inflammatory response-related genes were preserved as prognostic indicators, and the risk ratio of NOD2 gene was 2.07 (95% CI = 1.226-3.495, P = 0.006, **Figure 2C**). The correlation between these genes was presented in the **Figure 2D**.

### Construction of a Prognostic Model in the TCGA Cohort

**Figure 3:**
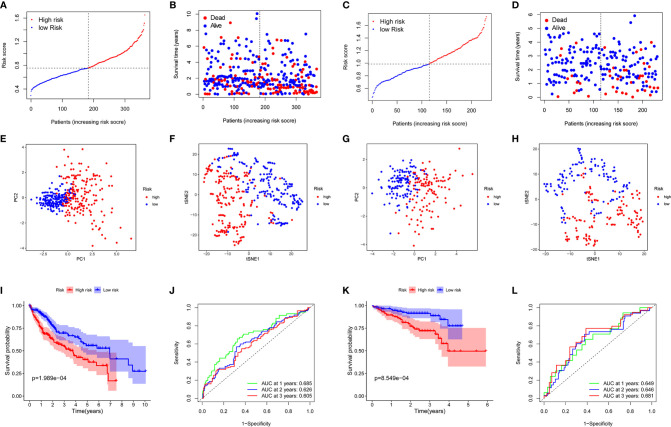
Prognostic analysis of the 8-gene signature model in the TCGA cohort and ICGC cohort. TCGA cohort **(A, B, E, F, I, J)**, ICGC cohort **(C, D, G, H, K, L)**. **(A, C)** The median value and distribution of the risk scores. **(B, D)** The distribution of OS status. **(E, G)** PCA plot. **(F, H)** t-SNE analysis. **(I, K)** Kaplan-Meier curves for OS of patients in the high- and low-risk groups. **(J, L)** AUC time-dependent ROC curves for OS.

The expression profiles of the above 13 genes were analyzed by LASSO-Cox regression analysis, and the prognostic model was established. A marker of eight genes was determined based on the optimal value of λ (**Supplementary Figure 1**). The risk score was calculated as follows: score = 0.118*expression level of SLC7A1 + 0.114*expression level of RIPK2 + 0.113*expression level of NOD2 + 0.022*expression level of ADORA2B+ 0.058*expression level of MEP1A+ 0.051*expression level of ITGA5 + 0.016*expression level of P2RX4 + 0.018*expression level of SERPINE1. Patients were divided into two groups according to the median cut-off value (**Figure 3A**). In the TCGA cohort, high-risk group was found to be significantly associated with higher tumor grade and advanced TNM stage (**Table 2**). PCA analysis and t-SNE analysis showed that patients in different risk groups were distributed in two directions (**Figures 3E–F**). Besides, the scatter chart indicated that patients with high risk were more likely to die earlier than those with low risk (**Figure 3B**). Consistently, the Kaplan-Meier curve showed the patients with high risk had a significantly worse OS than their low-risk counterparts (**Figure 3I**, P<0.001). Time-dependent ROC curves were generated for analysis of survival prediction by the prognostic model, and the area under the curve (AUC) reached 0.685 at 1 year, 0.626 at 2 years, and 0.605 at 3 years (**Figure 3J**). To explore the relationship between each prognostic gene and prognosis, survival analysis was performed based on the optimal cut-off expression value of each prognostic gene, which indicated that high expression of these genes was all significantly correlated with poor OS (**Supplementary Figures 2A–H**, P < 0.001). As shown in **Supplementary Figure 3**, the expression levels of all prognostic genes were higher in tumor tissues compared with adjacent non-tumorous tissues except SERPINE1.

**Table 2:** Baseline characteristics of the patients in different risk groups.

Characteristics	TCGA-LIHC cohort			ICGC-LIRP-JI cohort		
	High risk	Low risk	*P* value****	High risk	Low risk	*P* value
Age						
< 60 year	96 (26.3%)	69 (18.9%)	0.004	22 (9.5%)	22(9.5%)	0.975
≥ 60 year	86 (23.6%)	114 (31.2%)		94 (40.7%)	93 (40.3%)	
Gender						
Female	71 (19.5%)	48 (13.2%)	0.009	34 (14.7%)	27 (11.7%)	0.315
Male	111 (30.4%)	135 (37.0%)		82 (35.5%)	88 (38.1%)	
**Grade**						
G1+G2	97 (26.6%)	133 (36.4%)	<0.001	–	–	
G3+G4	82 (22.5%)	48 (13.2%)		–	–	
unknown	3 (0.8%)	2 (0.5%)		–	–	
Stage						
I + II	117 (32.1%)	137 (37.5%)	0.027	62 (26.8%)	79 (34.2%)	0.018
III + IV	52 (14.2%)	35 (9.6%)		54 (23.4%)	36 (15.6%)	
unknown	13 (3.6%)	11 (3.0%)		0	0	

### Validation of the 8-Gene Signature in the ICGC Cohort

To test the stability of the model constructed from the TCGA cohort, patients in the ICGC cohort were also categorized into high-risk or low-risk groups according to the median value from the TCGA cohort. Similar to the results obtained from the TCGA cohort, PCA and t-SNE analyses confirmed a discrete distribution of patients in the two subgroups (**Figures 3G, H**). Similarly, patients in the high-risk group were more likely to die earlier (**Figure 3D**) and had a shorter survival time compared with the low-risk group (**Figure 3K**). Besides, the AUC of the 8-gene signature was 0.649 at 1 year, 0.646 at 2 years, and 0.681 at 3 years (**Figure 3L**).

### Independent Prognostic Value of the 8-Gene Signature

**Figure 4:**
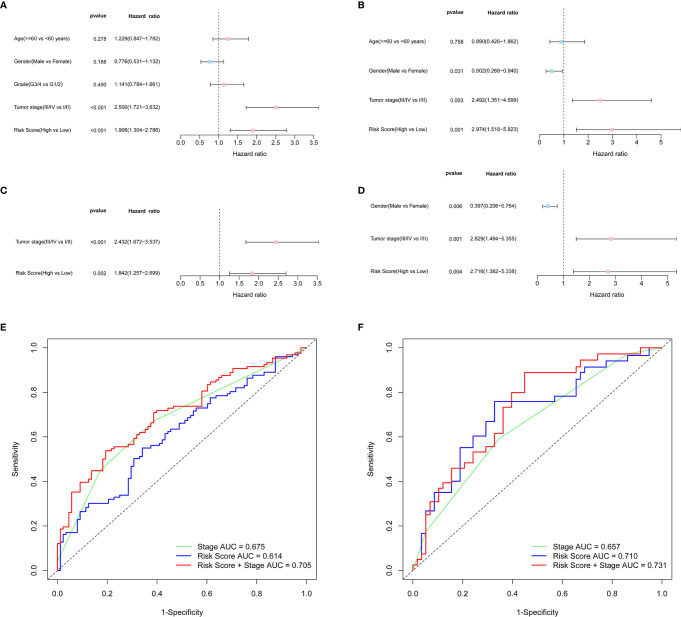
OS-related factors were screened, and the prognostic accuracy of risk score and clinicopathological factors were compared. TCGA cohort **(A, C, E)**, ICGC cohort **(B, D, F)**. **(A, B)** OS-related factors were screened by Univariate Cox regression analyses. **(C, D)** OS-related factors were screened by Multivariate Cox regression analysis. **(E, F)** Time-dependent ROC curve was used to compare the prognostic accuracy of risk score, tumor stage, and the combination of risk score and tumor stage in 3-year.

Univariate and multivariate Cox analyses of variables were employed to determine whether the risk score was an independent prognostic factor for OS. In univariate Cox analysis, the risk scores in both TCGA and ICGC cohorts were significantly correlated with OS (TCGA cohort: HR = 1.906, 95% CI = 1.304-2.786, P < 0.001; ICGC cohort: HR = 2.974, 95% CI = 1.518-5.823, P = 0.001) (**Figures 4A, B**). After correcting for other confounding factors, multivariate Cox analysis showed that the risk score was still an independent predictor for OS (TCGA cohort: HR = 1.842, 95% CI = 1.257-2.699, P = 0.002; ICGC cohort: HR = 2.716, 95% CI = 1.382-5.338, P = 0.004) (**Figures 4C, D**). ROC curve analysis showed that the risk score had good predictive accuracy of prognosis, and it combined with tumor stage provided a more accurate prediction in 3-year OS in HCC patients, wherever in TCGA dataset (AUC = 0.705) or in the ICGC dataset (AUC = 0.731) (**Figures 4E, F**). Therefore, the combination of risk score and clinicopathological features had excellent prognostic value of HCC.

### Prognostic Model Risk Score and Clinical Features

**Figure 5:**
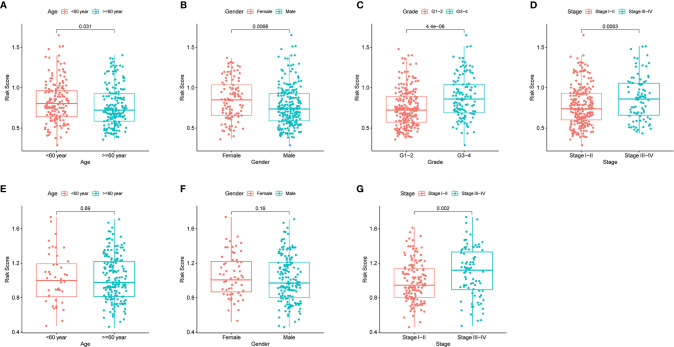
The risk score in different groups divided by clinical characteristics. TCGA cohort **(A–D)**, ICGC cohort **(E–F)**. **(A, E)** Age. **(B, F)** Gender. **(C)** Tumor grade. **(D, G)** Tumor stage.

By analyzing the association of risk score with the clinical characteristics of HCC patients, we showed that the risk score was significantly higher in tumor grade 3-4 (P < 0.001) or tumor stage III-IV (P < 0.01) compared with tumor grade 1-2 or tumor stage I-II (**Figures 5C, D**). In addition, the same analysis in the ICGC dataset confirmed that the risk score was definitely higher in tumor stage III-IV compared with tumor stage I-II (There was no data about the grade of HCC in the ICGC dataset) (**Figure 5G**). Furthermore, the results revealed that the expression of prognostic genes was significantly higher in tumor grade 3-4 compared with tumor grade 1-2 except SERPINE1 (P < 0.05, **Supplementary Figure 4C**). The expression of ADORA2B, SERPINE1 and SLC7A1 was definitely higher in tumor stages III-IV compared with tumor stage I-II (P < 0.05, **Supplementary Figure 4D**). In addition, the expression of ITGA5 and SLC7A1 was different between age <=60 year and age > 60 year, and the expression of ITGA5, MEP1A, RIPK2 and SLC7A1 were different between female and male (P < 0.05, **Supplementary Figures 4A, B**).

### Immune Status and Tumor Microenvironment Analysis

**Figure 6:**
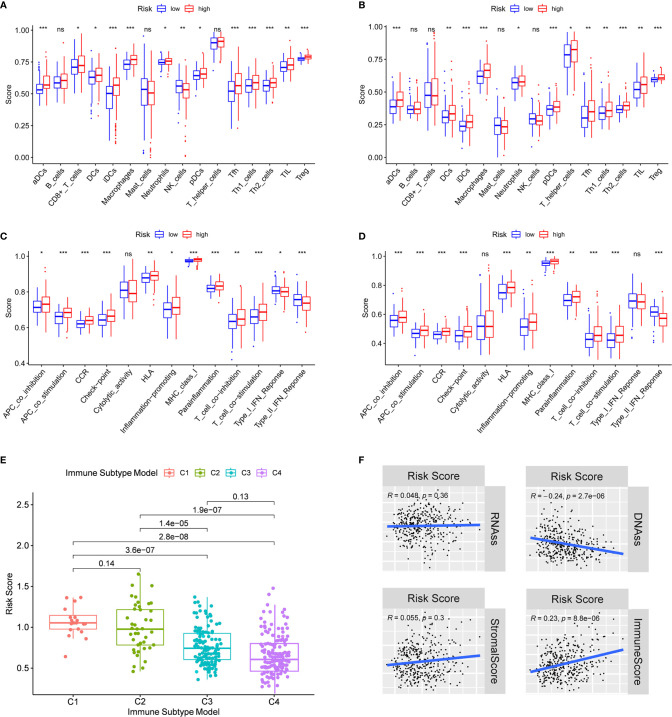
Immune status between different risk groups and the association between risk score and tumor microenvironment. TCGA cohort **(A, C)**, ICGC cohort **(B, D)**. **(A, B)** The scores of 16 immune cells and **(C, D)** 13 immune-related functions were showed in boxplots. **(E)** Comparison of the risk score in different immune infiltration subtypes. **(D)** The relationship between risk score and RNAss, DNAss, Stromal Score and Immune Score. P values were showed as: ns, not significant; *P < 0.05; **P < 0.01; ***P < 0.001.

In order to further explore the correlation between risk score and immune status, the enrichment scores of different immune cell subpopulations, related functions and pathways were quantified by ssGSEA. We found that the contents of the antigen presentation process in the TCGA cohort, including aDCs, iDCs, pDCs, APC co-inhibition, APC co-stimulation, HLA and MHC class I, were significantly elevated in the high-risk group (all adjusted P < 0.05, **Figures 6A, C**). In addition, compared with the low-risk group, the fractions of Tfh cells, Treg cells, Th1 cells, Th2 cells, T cell co-stimulation and T cell co-inhibition were higher in high-risk group, indicating the differences in T cell regulation between high- and low-risk groups. Furthermore, the scores of CCR, check-point, macrophages, neutrophils and inflammation-promoting activity were higher in the high-risk group, while the activity of type II IFN response score was just the opposite (adjusted P < 0.05). The results of comparisons in the ICGC cohort were similar to those in the TCGA between the two risk groups (adjusted P < 0.05, **Figures 6B, D**).

To understand how risk score was associated with immune components, we tested the correlation between risk score and immune infiltrates. Six types of immune infiltrates were identified in human tumors, which corresponded from tumor promoting to tumor suppressing respectively (17), namely C1 (wound healing), C2 (INF-γ dominant), C3 (inflammatory), C4 (lymphocyte depleted), C5 (immunologically quiet) and C6 (TGF-β dominant) [29]. No patient sample belonged to C5 immune subtype in HCC and only 1 sample belonged to C6 immune subtype, so C5 and C6 immune subtypes were not included in the study. We analyzed the immune infiltration of HCC in TCGA-HCC data and correlated it with risk score, and the results showed that high risk score was significantly associated with C1, while low risk score was significantly associated with C4 (**Figure 6E**). As shown in **Supplementary Figure 5**, except for NOD2, the high expression of prognostic genes was significantly associated with C1. On the contrary, the expression of all prognostic genes was definitely associated with C4.

**Figure 7:**
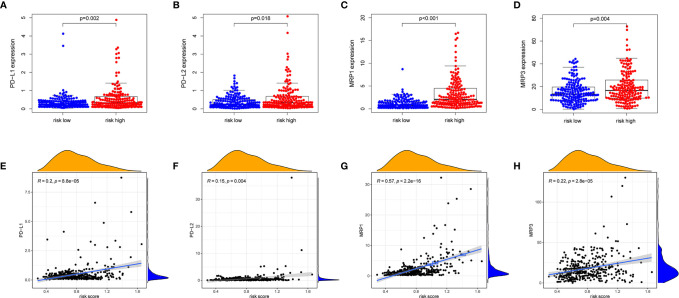
The comparison of the expression levels of PD-L1, PD-L2, MRP1 and MRP3 between different risk groups and correlation analysis between risk score and the expression levels of PD-L1, PD-L2, MRP1 and MRP3. **(A, E)** PD-L1. **(B, F)** PD-L2. **(C, G)** MRP1. **(D, H)** MRP3.

PD-1/PD-L1 and PD-1/PD-L2 pathways are key regulators in cancer immune evasion. The expression levels of immune checkpoints including PD-L1 and PD-L2 are important indicators for individualized immunotherapy. As expected, the expression levels of PD-L1 and PD-L2 were significantly higher in the high-risk group compared with the low-risk group (**Figures 7A, B**) and the expression levels of these immune checkpoints showed a positive correlation with the risk score (**Figures 7E, F**). In terms of tumor drug resistance genes, high-risk group had higher expression of MRP1 and MRP3 compared with low-risk group (**Figures 7C, D**). Furthermore, the expression of MRP1 and MRP3 was significantly positively correlated with risk score (**Figures 7G, H**).

Tumor stemness can be measured by RNA stemness score (RNAss) based on mRNA expression and DNA stemness score based on DNA methylation pattern (DNAss) (18). Stromal score and immune score were used to estimate tumor immune microenvironment. The correlation analysis was performed to explore whether the risk score was associated with tumor stem cells and the immune microenvironment, and the results indicated that the risk score was not significantly associated with DNAss and RNAss, but significantly positively correlated with immune score (P < 0.001) (**Figure 6F**). Besides, the correlation between prognostic gene expression and tumor stem cells was analyzed, and the results showed that ITGA5 and SCL7A1 were significantly negatively correlated with RNAss and DNAss. MEP1A and P2RX4 were significantly positively correlated with RNAss (**Supplementary Figure 6**). Since stromal cells were the important components of the tumor microenvironment, especially in HCC, we further investigated the correlation between immune microenvironment and prognostic gene expression. We found that ITGA5, NOD2, SERPINE2 and SLC7A1 were positively correlated with the stromal score of HCC, suggesting that ITGA5, NOD2, SERPINE2 and SLC7A1 were expressed in the stroma of HCC tissues. In addition, ITGA5, NOD2, P2RX4, RIPK2, SERPINE1 and SLC7A1 were all significantly positively correlated with the immune score, which measured the presence of infiltrating immune cells.

### Biological Function and Pathway Analyses

**Figure 8:**
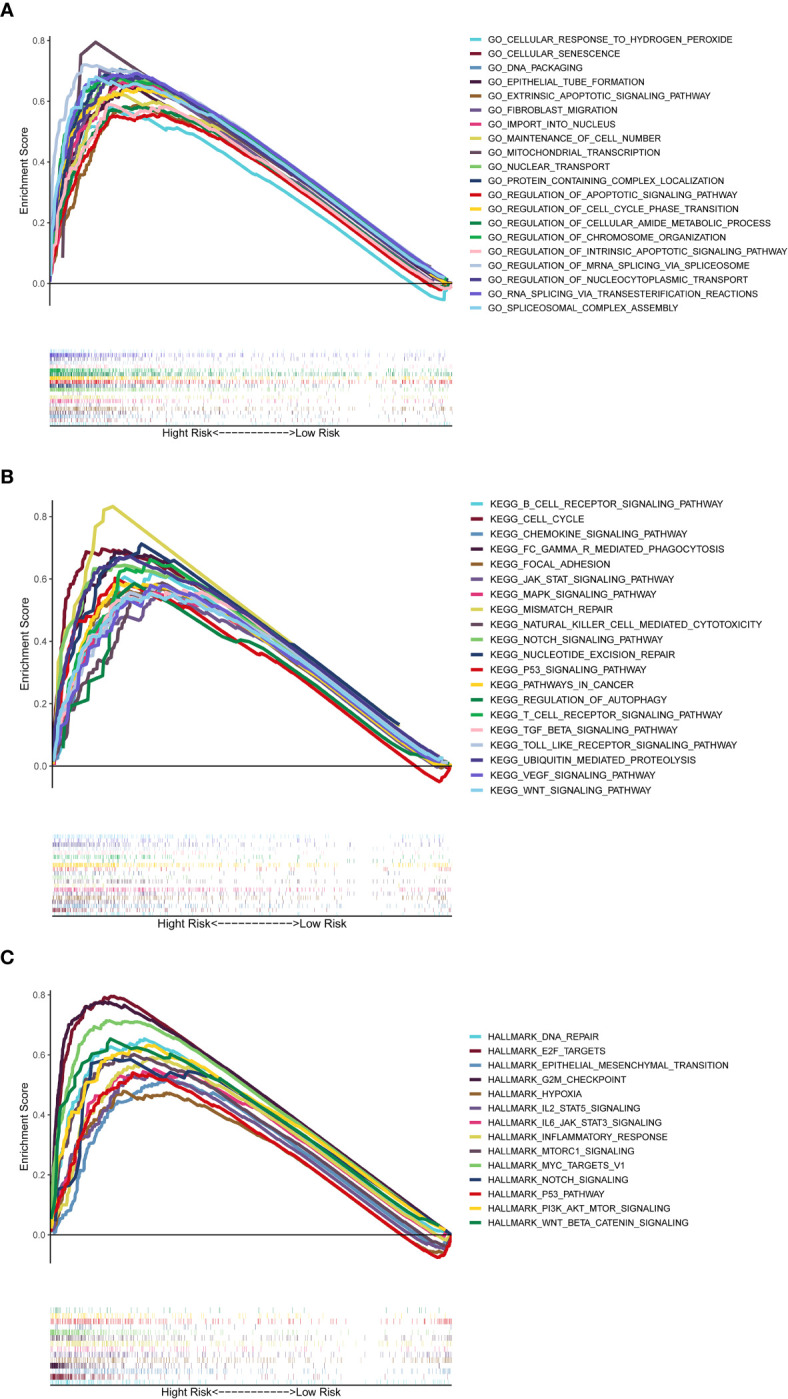
Gene set enrichment analysis of Biological functions and pathways. **(A)** GO, Gene Ontology. **(B)** KEGG, Kyoto Encyclopedia of Genes and Genomes. **(C)** Hallmark gene set.

The GSEA was used to perform GO function and KEGG pathway enrichment analyses between the high- and low-risk groups. GO function enrichment analysis revealed that regulation of cell cycle phase transition was significantly enriched in the high-risk group (**Figure 8A**, **Supplementary Figure 7**). Besides, 20 KEGG pathways were enriched in the high-risk group with a false discovery rate < 0.05 (**Figure 8B**, **Supplementary Figure 8**). The results revealed that some pathways related to cancer process such as Cell Cycle, JAK-STAT, MAPK, NOTCH, P53 and WNT were enriched. In addition, the KEGG pathways also included the Chemotaxis, Fc-γ receptor mediated phagocytosis, T cell receptor and Toll-like receptor, which were correlative with inflammatory response. Similar to the results of KEGG, GSEA using TCGA data of the Hallmarks gene sets indicated that NOTCH, P53, IL2-Stat5-Signaling, IL6-Jak-Stat3-Signaling and Inflammatory Response pathways were statistically significant programs (**Figure 8C**, **Supplementary Figure 9**).

### Prognostic Gene Expression and Cancer Cell Sensitivity to Chemotherapy

**Figure 9:**
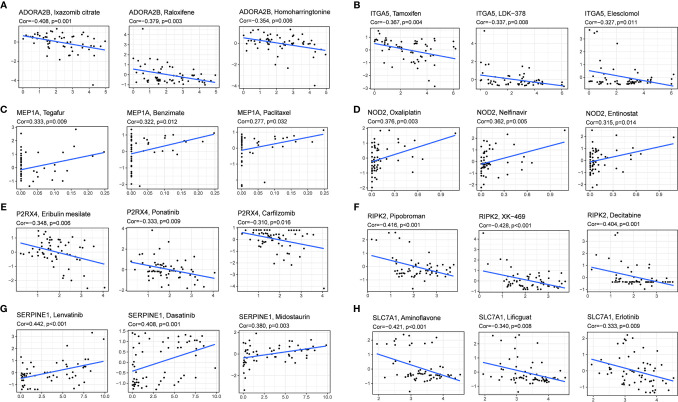
Scatter plot of relationship between prognostic gene expression and drug sensitivity. **(A)** ADORA2B. **(B)** ITGA5. **(C)** MEP1A. **(D)**NOD2. **(E)** P2RX4. **(F)** RIPK2. **(G)** SERPINE1. **(H)** SLC7A1.

We investigated the expression of prognostic genes in NCI-60 cell lines and analyzed the relationship between their expression levels and drug sensitivity. The results showed that all prognostic genes were correlative to some chemotherapy drug sensitivity (P < 0.01) (**Figure 9**). For example, increased expression of ADORA2B, SLC7A1, ITGA5, RIPK2 and P2RX4 was associated with increased drug resistance of cancer cells to Ixazomib citrate, Homoharringtonine, Erlotinib, Tamoxifen, Elesclomol, LDK-378, Pipobroman, Decitabine, eribulin mesylate, ponatinib, carfilzomib, etc. On the contrary, increased expression of NOD2 and MEP1A was associated with increased drug sensitivity of cancer cells to a number of chemotherapy drugs such as Oxaliplatin, Nelfinavir, Entinostat, Tegafur, Benzimate and Paclitaxel. Interestingly, increased expression of SERPINE1 was associated with increased drug sensitivity of cancer cells to Lenvatinib, which was approved by the FDA as the first-line treatment for unresectable HCC in 2018.

### Verification of the Prognostic Gene Expression Between HCC Tissues and Adjacent Non-Tumorous Tissues by qRT-PCR and IHC

**Figure 10:**
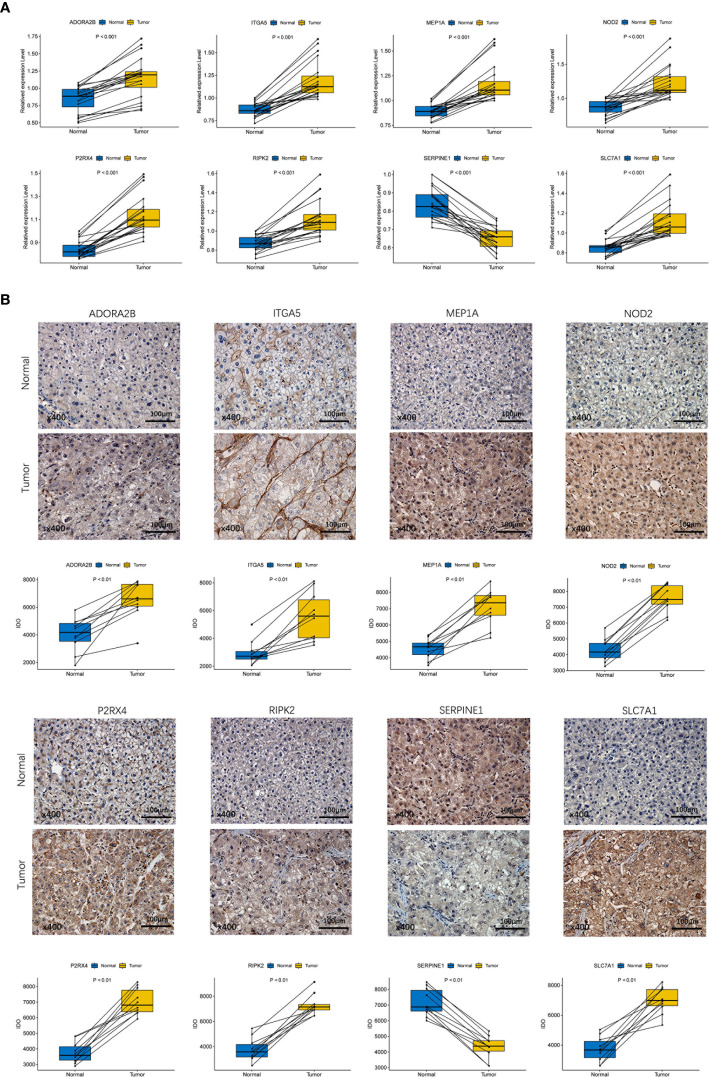
Experiment confirmed the difference of the prognostic gene expression between HCC and adjacent non-tumor tissues. **(A)** The mRNA expression analysis by qRT-RCR. **(B)** The protein expression analysis by IHC.

To validate the different expression of the eight prognostic genes (ADORA2B, MEP1A, P2RX4, SERPINE1, ITGA5, NOD2, RIPK2 and SLC7A1) between HCC tissues and adjacent non-tumorous tissues, qRT-PCR and IHC were implemented to analyze the mRNA and protein expression respectively. The results of qRT-PCR showed that prognostic genes except SERPINE1 were highly expressed in HCC tissues compared with adjacent non-tumorous tissues (**Figure 10A**, P < 0.001). IHC staining showed the same results as qRT-PCR (**Figure 10B**, P < 0.01). The validation results were consistent with RNA sequencing expression of eight prognostic genes in the TCGA dataset (**Supplementary Figure 3**).

## Discussion

With the establishment of next-generation sequencing technology and the beginning of the era of precision medicine, various treatments for HCC have been developed. However, we are often unable to make early diagnosis and predict the therapeutic effect of HCC due to the small number of useful biomarkers. Previous studies indicated that novel serum biomarkers including circulating tumor cells, circulating nucleic acids (19), and the combination of retinol and retinal panel (20) have excellent accuracy of HCC prognosis. In addition, inflammatory response-related serum biomarkers such as medium-granulocyte ratio, platelet-lymphoid ratio and lymphoid-monocyte ratio also have a good performance in predicting prognosis of HCC (10). However, the inflammatory response-related gene signature as prognostic marker for HCC has not been reported. Previous studies indicated that ferroptosis-related gene signature, immune-related gene signature, energy metabolism-related gene signature, m6A-related gene signature and hypoxia-related gene signature predict 3-year OS for HCC with AUC at 0.668, 0.663, 0.69, 0.647 and 0.685 (21–25), respectively, which were similar to our research. In addition to good predictive performance for HCC prognosis, the inflammatory response-related gene signature constructed in our study demonstrates more advantages compared with gene signatures above. For example, it can distinguish immune checkpoints genes and tumor drug resistance genes to a high-expression group and a low-expression group, and risk score has been proved to be correlated with many chemotherapeutic drugs resistance. A recent study pointed out that mSEPT9 as a prognostic marker of HCC has remarkable predictive effect for prognosis in HCC (AUC = 0.85) (26). The methylation level of SEPT9 gene was detected by methylation specific PCR (MS-PCR). MS-PCR can only detect a few methylation sites in the gene sequence. However, the gene methylation sites are widely distributed in the DNA sequence, so there is a certain deviation in the methylation level of the whole gene when the MS-PCR results are used to represent the methylation level of the whole gene. However, in our study, the expression levels of genes in prognostic signature were determined by high-throughput sequencing, a frequently used technique that could provide accurate results.

In this study, we systematically analyzed the expression of 200 inflammatory response-related genes in HCC tissues and their relationship with OS. Forty-four DEGs were screened out from the TCGA cohort. Univariate Cox analysis showed that 14 of DEGs were associated with OS. A prognostic model integrating 8 inflammatory response-related genes was constructed by LASSO regression analysis and validated in the ICGC cohort. According to the median risk score, patients were divided into high- and low-risk groups. We found that high-risk group was significantly correlated with higher tumor grade, advanced TNM stage and shorter OS period. Independent prognostic analysis showed that risk score was an independent predictor for OS.

The prognostic model established in this study consisted of 8 inflammatory response-related genes (ADORA2B, ITGA5, MEP1A, NOD2, P2RX4, RIPK2, SERPINE1 and SLC7A1). These genes were all upregulated in HCC tumor tissues and associated with poor prognosis except SERPINE1. ADORA2B is an adenosine A2B receptor, which was reported to play an important role in tumorigenesis and development by regulating immune system and modulating proliferation, differentiation and apoptosis of parenchymal cells (27). Sorafenib combined with adenosine A receptor blocker significantly reduced the progression of hepatoma in mice (28). Integrin family including ITGA5, expressed by tumor and tumor-related host cells, mediates a variety of cellular effects, leading to tumor progression and metastasis (29). MEP1A has been explored as a prognostic marker for patients with HCC, especially early HCC, and it may play an important role in the progression of HCC by promoting migration and invasion of cancer cells (30). P2X4 receptor may be closely related to the downstream inflammatory process by activation of oxidative stress, inflammasome, and immune modulation for continuous cancer progression (31). Arun Asif et al. demonstrated that increased RIPK2 activity leads to the activation of NF-κB, which up-regulates the proliferation, invasion, metastasis and anti-apoptosis of cancer cells (32). By inhibiting proteolytic activity and promoting angiogenesis, increased expression of SERPINE1 in colon cancer models may lead to the spread of malignant tumors (33), and high expression of SERPINE1 is a poor prognostic indicator of breast cancer (34). However, contrary to expectations, SERPINE1 had lower expression in HCC tumor tissues than adjacent normal tissues in TCGA dataset. It seemed to contradict the result that the high expression of SERPINE1 in cancer indicates poor survival, which may be explained by the following reason that SERPINE1 serves as different roles in tumor and normal tissues. Compared with HCC, the relatively high expression of SERPINE1 in normal liver tissue is essential for maintaining cell growth. On the contrary, high expression of SERPINE1 acts as tumor promoter in tumor tissues due to interact with some tumorigenic factors, resulting in poor prognosis. But its idiographic action mechanism remains to be addressed.

To gain more insight into the relationship between risk score and immune components, we studied the role of risk score in immune infiltration type. Interestingly, we showed that high risk score was significantly correlated with C1, while low risk score was definitely associated with C4, indicating that C1 promotes the occurrence and development of tumor and C4 is a good protective factor. This discovery was consistent with the results of previous studies, because high cytotoxicity can inhibit the occurrence and development of tumor (17). In terms of the association between risk score and clinical characteristics, high risk score was significantly associated with tumor grade 3-4 or tumor stage III-IV, which indicated that high risk score is definitely related with poor prognosis.

However, whether these genes affect the prognosis of HCC patients by inflammatory response remains to be elucidated, because there were few studies on these genes. Based on the GSEA analysis, tumor-related signal pathways such as JAK-STAT, MAPK, p53 and NOTCH were significantly enriched, and continuous activation of these pathways has been confirmed to be linked with HCC, which would be new therapeutic targets (35–38). Inflammation-related signal pathways such as Chemotaxis, Fc-γ receptor-mediated phagocytosis, T cell receptor, Toll-like receptor, IL2-Stat5-Signaling, IL6-Jak-Stat3-Signalling and Inflammatory Response pathways were significantly enriched in the high-risk group, which further validated that the inflammatory response has a close connection with tumor procession. Besides, high-risk group had higher fractions of macrophages, neutrophils and Treg cells. Previous studies have demonstrated that the increase of tumor-associated macrophages (39, 40), neutrophils (39) and Treg cells (39, 41) is associated with poor prognosis in patients with HCC due to their role in immune invasion. Cancer immunotherapies that target immune checkpoints such as anti-PD-L1 antibodies have shown clinical activity in various cancer types (42). Increased immune checkpoint suppresses the anti-tumor immune response of T cells by increasing the expression of PD-1 and CTLA4 receptors, and research on immune checkpoint inhibitors has made significant progress in the treatment of HCC (43). In our study, the score of immune checkpoints in the high-risk group was higher compared with the low-risk group and risk score was positively correlated with the expression of PD-L1 and PD-L2. Therefore, the prognostic model can predict the expression level of immune checkpoints and have the potential to guide immunotherapy decisions. In addition, the high risk score was related to the impairment of activity of type II IFN response, which plays an important role in tumor immune surveillance, stimulating anti-tumor immunity and promoting tumor elimination (44–49). Moreover, increased activities of Tfh cells, Treg cells, Th1 cells, Th2 cells, T cell co-stimulation and T cell co-inhibition in the high-risk group indicated that immune regulatory function in the high-risk group is disturbed. Therefore, it is reasonable to assume that anti-tumor immunity of the high-risk group is attenuated, which may be an important reason for its poor prognosis.

At present, cancer biology is constantly changing from a “cancer cell-centered” view to a more inclusive concept, in which cancer cells are placed in a network of stromal cells made up of fibroblasts, vascular cells and inflammatory immune cells. These cells make up the tumor microenvironment (5). Cancer stem cell-like cells (CSCs) can be derived from different sources, including long-lived stem cells, progenitor cells or converting from non-stem cells through dedifferentiation (18). CSCs promote tumor progression due to their ability of self-renewal and invasion, which is the main cause of treatment induced drug resistance (50–52). The correlation between prognostic gene expression and tumor stem cell score suggested that ITGA5 and SLC7A1 may have a tumor inhibitory effect, because they were negatively correlated with tumor stemness based on DNAss and RNAss. However, this conclusion is contrary to the role of ITGA5 in tumors (53–55), which may be because ITGA5 plays the opposite role through different pathways. It is possible that ITGA5 inhibits differentiation of tumor stem cells but promotes tumor proliferation and invasion, of which the specific mechanism is worthy of further study. According to ESTIMATE algorithm, the prognostic gene expression was also correlated with stromal score and immune score to some extent. There was a strong correlation between ITGA5, NOD2, SERPINE1, SLC7A1 and stromal score, suggesting that they may be secreted by stromal cells or participate in stroma related activities. And the positive correlation between ITGA5, NOD2, P2RX4, RIPK2, SLC7A1 and immune score indicated that the tumor tissue in the high-risk group is highly infiltrated by immune cells, which is consistent with risk score.

Using NCI-60 cell lines data, we found that increased expression of some prognostic genes was associated with increased drug resistance for a number of FDA approved chemotherapy drugs, such as Tamoxifen, Lxazomib citrate, Pipobroman, Homoharringtonine and Decitabine. Of course, various prognostic genes were also associated with increased drug sensitivity of a few drugs. For instance, increased expression of SERPINE1 was associated with sensitivity of cancer cells to Lenvatinib, which was approved by the FDA as the first-line treatment for unresectable HCC in 2018. The MRP family comprises 13 members, among which MRP1 to MRP9 are the main transporters indicated to result in multidrug resistance by extruding anticancer drugs out of tumor cells (56). Hence, the correlation between risk score and drug resistance genes including MRP1 and MRP3 suggested that targeting tumor drug resistance genes appears to have a therapeutic potential for high-risk patients. These data demonstrated that some prognostic genes can be used as therapeutic targets to overcome drug resistance or adjuvant drug sensitivity.

## Conclusion

To sum up, our study defined a new prognostic signature consisting of eight inflammatory response-related genes. The signature was proved to be independently associated with OS in TCGC cohort and ICGC validation cohort, and was confirmed to be valuable in functional analysis, tumor microenvironment and drug sensitivity, providing insight for predicting the prognosis of HCC. The specific potential mechanism between inflammatory response-related genes and tumor immunity in HCC remains unclear, which is worthy of further study. Taken together, our work will go a long way towards revealing their role in tumorigenesis, particularly in the areas of immune response, tumor microenvironment and drug resistance, which is essential for the development of personalized cancer therapies.

## Data Availability Statement

The original contributions presented in the study are included in the article/**Supplementary Material**. Further inquiries can be directed to the corresponding authors.

## Ethics Statement

The studies involving human participants were reviewed and approved by Human Research Ethics Committee in The First Affiliated Hospital of Wenzhou Medical University. The patients/participants provided their written informed consent to participate in this study. Written informed consent was obtained from the individual(s) for the publication of any potentially identifiable images or data included in this article.

## Author Contributions

FY and ZL conceived and designed the study. ZL and QX provided equal contributions to research design, data analysis, and article writing. DM revised the manuscript. All authors contributed to the article and approved the submitted version.

## Funding

The project was supported by Zhejiang Provincial Natural Science Foundation of China (No. LY19H030005), the National Natural Science Foundation of China (No. 81970527/H0317), Wenzhou Municipal Science and technology Bureau (No. 2018Y0064).

## Conflict of Interest

The authors declare that the research was conducted in the absence of any commercial or financial relationships that could be construed as a potential conflict of interest.

## Supplementary Material

Supplementary Table 1 The 200 inflammatory response-related genes

Supplementary Table 2 The 263 chemotherapy drugs of FDA approved or on clinical trials.

Supplementary Table 3 The primer sequences of the eight prognostic genes.

Supplementary Table 4 The results of correlation analysis of the prognosis gene expression and chemotherapy drug sensitivity.

Supplementary Table 5 The information of primary antibodies used in IHC.

Supplementary Figure 1 Constructed an 8-gene signature in the TCGA cohort. **(A)** LASSO coefficient expression profiles of 13 candidate genes. **(B)** The penalty parameter (λ) in the LASSO model was selected through ten cross-validation.

Supplementary Figure 2 Survival analysis of prognostic genes according to the optimal cut-off expression value. TCGA cohort **(A–H)**. All adjusted P < 0.05.

Supplementary Figure 3 Each prognostic gene expression between HCC tissues and adjacent non-tumorous tissues in TCGA.

Supplementary Figure 4 Each prognostic gene expression in different groups divided by clinical characteristics. **(A)** Age. **(B)** Gender. **(C)** Tumor grade. **(D)** Tumor stage.

Supplementary Figure 5 The expression of prognostic genes in different immune infiltrate subtypes.

Supplementary Figure 6 The relationship of prognostic gene expression with RNAss, DNAss, Stromal Score and Immune Score.

Supplementary Figure 7 Significant GO terms associated with high-risk score group.

Supplementary Figure 8 Significant KEGG pathways associated with high-risk score group.

Supplementary Figure 9 Significant Hallmarks gene sets associated with high-risk score group.

## References

[1] Bray F Ferlay J Soerjomataram I Siegel RL Torre LA Jemal A. Global cancer statistics 2018: GLOBOCAN estimates of incidence and mortality worldwide for 36 cancers in 185 countries. CA Cancer J Clin (2018) 68(6):394–424. 10.3322/caac.21492 30207593

[2] Yang JD Hainaut P Gores GJ Amadou A Plymoth A Roberts LR. A global view of hepatocellular carcinoma: trends, risk, prevention and management. Nat Rev Gastroenterol Hepatol (2019) 16(10):589–604. 10.1038/s41575-019-0186-y 31439937 PMC6813818

[3] Allemani C Matsuda T Di Carlo V Harewood R Matz M Niksic M. Global surveillance of trends in cancer survival 2000-14 (CONCORD-3): analysis of individual records for 37 513 025 patients diagnosed with one of 18 cancers from 322 population-based registries in 71 countries. Lancet (2018) 391(10125):1023–75. 10.1016/S0140-6736(17)33326-3 PMC5879496 29395269

[4] Balkwill F Mantovani A. Inflammation and cancer: back to Virchow? Lancet (2001) 357(9255):539–45. 10.1016/S0140-6736(00)04046-0 11229684

[5] Greten FR Grivennikov SI. Inflammation and Cancer: Triggers, Mechanisms, and Consequences. Immunity (2019) 51(1):27–41. 10.1016/j.immuni.2019.06.025 31315034 PMC6831096

[6] Coussens LM Werb Z. Inflammation and cancer. Nature (2002) 420(6917):860–7. 10.1038/nature01322 PMC2803035 12490959

[7] Grivennikov SI Greten FR Karin M. Immunity, inflammation, and cancer. Cell (2010) 140(6):883–99. 10.1016/j.cell.2010.01.025 PMC2866629 20303878

[8] Shalapour S Karin M. Pas de Deux: Control of Anti-tumor Immunity by Cancer-Associated Inflammation. Immunity (2019) 51(1):15–26. 10.1016/j.immuni.2019.06.021 31315033 PMC6640850

[9] Sanghera C Teh JJ Pinato DJ. The systemic inflammatory response as a source of biomarkers and therapeutic targets in hepatocellular carcinoma. Liver Int (2019) 39(11):2008–23. 10.1111/liv.14220 31433891

[10] Yu JI Park HC Yoo GS Paik SW Choi MS Kim HS. Clinical Significance of Systemic Inflammation Markers in Newly Diagnosed, Previously Untreated Hepatocellular Carcinoma. Cancers (Basel) (2020) 12(5). 10.3390/cancers12051300 PMC7281027 32455607

[11] McMillan DC. The systemic inflammation-based Glasgow Prognostic Score: a decade of experience in patients with cancer. Cancer Treat Rev (2013) 39(5):534–40. 10.1016/j.ctrv.2012.08.003 22995477

[12] Budhu A Forgues M Ye QH Jia HL He P Zanetti KA. Prediction of venous metastases, recurrence, and prognosis in hepatocellular carcinoma based on a unique immune response signature of the liver microenvironment. Cancer Cell (2006) 10(2):99–111. 10.1016/j.ccr.2006.06.016 16904609

[13] Simon N Friedman J Hastie T Tibshirani R. Regularization Paths for Cox’s Proportional Hazards Model via Coordinate Descent. J Stat Software (2011) 39(5):1–13. 10.18637/jss.v039.i05 PMC4824408 27065756

[14] Tibshirani R. The lasso method for variable selection in the Cox model. Stat Med (1997) 16(4):385–95. 10.1002/(SICI)1097-0258(19970228)16:4<385::AID-SIM380>3.0.CO;2-3 9044528

[15] Yoshihara K Shahmoradgoli M Martinez E Vegesna R Kim H Torres-Garcia W. Inferring tumour purity and stromal and immune cell admixture from expression data. Nat Commun (2013) 4:2612. 10.1038/ncomms3612 24113773 PMC3826632

[16] Dib L San-Jose LM Ducrest AL Salamin N Roulin A. Selection on the Major Color Gene Melanocortin-1-Receptor Shaped the Evolution of the Melanocortin System Genes. Int J Mol Sci (2017) 18(12). 10.3390/ijms18122618 PMC5751221 29206201

[17] Tamborero D Rubio-Perez C Muinos F Sabarinathan R Piulats JM Muntasell A. A Pan-cancer Landscape of Interactions between Solid Tumors and Infiltrating Immune Cell Populations. Clin Cancer Res (2018) 24(15):3717–28. 10.1158/1078-0432.CCR-17-3509 29666300

[18] Malta TM Sokolov A Gentles AJ Burzykowski T Poisson L Weinstein JN. Machine Learning Identifies Stemness Features Associated with Oncogenic Dedifferentiation. Cell (2018) 173(2):338–54.e15. 10.1016/j.cell.2018.03.034 29625051 PMC5902191

[19] Trevisan Franca de Lima L Broszczak D Zhang X Bridle K Crawford D Punyadeera C. The use of minimally invasive biomarkers for the diagnosis and prognosis of hepatocellular carcinoma. Biochim Biophys Acta Rev Cancer (2020) 1874(2):188451. 10.1016/j.bbcan.2020.188451 33065194

[20] Han J Han ML Xing H Li ZL Yuan DY Wu H. Tissue and serum metabolomic phenotyping for diagnosis and prognosis of hepatocellular carcinoma. Int J Cancer (2020) 146(6):1741–53. 10.1002/ijc.32599 31361910

[21] Liang JY Wang DS Lin HC Chen XX Yang H Zheng Y. A Novel Ferroptosis-related Gene Signature for Overall Survival Prediction in Patients with Hepatocellular Carcinoma. Int J Biol Sci (2020) 16(13):2430–41. 10.7150/ijbs.45050 PMC7378635 32760210

[22] Dai Y Qiang W Lin K Gui Y Lan X Wang D. An immune-related gene signature for predicting survival and immunotherapy efficacy in hepatocellular carcinoma. Cancer Immunol Immunother (2020). 10.1007/s00262-020-02743-0 PMC10992402 33089373

[23] Li Z Li F Peng Y Fang J Zhou J. Identification of three m6A-related mRNAs signature and risk score for the prognostication of hepatocellular carcinoma. Cancer Med (2020) 9(5):1877–89. 10.1002/cam4.2833 PMC7050095 31943856

[24] Chen Q Li F Gao Y Xu G Liang L Xu J. Identification of Energy Metabolism Genes for the Prediction of Survival in Hepatocellular Carcinoma. Front Oncol (2020) 10:1210. 10.3389/fonc.2020.01210 32903581 PMC7438573

[25] Jiang HY Ning G Wang YS Lv WB. Ahypoxia-related signature enhances the prediction of the prognosis in hepatocellular carcinoma patients and correlates with sorafenib treatment response. Am J Transl Res (2020) 12(12):7762–81. PMC7791514 33437359

[26] Song L Chen Y Gong Y Wan J Guo S Liu H. Opportunistic screening and survival prediction of digestive cancers by the combination of blood mSEPT9 with protein markers. Ther Adv Med Oncol (2020) 12:1758835920962966. 10.1177/1758835920962966 33403008 PMC7745555

[27] Antonioli L Blandizzi C Pacher P Hasko G. Immunity, inflammation and cancer: a leading role for adenosine. Nat Rev Cancer (2013) 13(12):842–57. 10.1038/nrc3613 24226193

[28] Liao J Zeng DN Li JZ Hua QM Xiao Z He C. Targeting adenosinergic pathway enhances the anti-tumor efficacy of sorafenib in hepatocellular carcinoma. Hepatol Int (2020) 14(1):80–95. 10.1007/s12072-019-10003-2 31802389

[29] Desgrosellier JS Cheresh DA. Integrins in cancer: biological implications and therapeutic opportunities. Nat Rev Cancer (2010) 10(1):9–22. 10.1038/nrc2748 20029421 PMC4383089

[30] OuYang HY Xu J Luo J Zou RH Chen K Le Y. MEP1A contributes to tumor progression and predicts poor clinical outcome in human hepatocellular carcinoma. Hepatology (2016) 63(4):1227–39. 10.1002/hep.28397 26660154

[31] Asif A Khalid M Manzoor S Ahmad H Rehman AU. Role of purinergic receptors in hepatobiliary carcinoma in Pakistani population: an approach towards proinflammatory role of P2X4 and P2X7 receptors. Purinergic Signal (2019) 15(3):367–74. 10.1007/s11302-019-09675-0 PMC6737133 31401785

[32] Zare A Petrova A Agoumi M Amstrong H Bigras G Tonkin K. RIPK2: New Elements in Modulating Inflammatory Breast Cancer Pathogenesis. Cancers (Basel) (2018) 10(6). 10.3390/cancers10060184 PMC6025367 29874851

[33] Mazzoccoli G Pazienza V Panza A Valvano MR Benegiamo G Vinciguerra M. ARNTL2 and SERPINE1: potential biomarkers for tumor aggressiveness in colorectal cancer. J Cancer Res Clin Oncol (2012) 138(3):501–11. 10.1007/s00432-011-1126-6 PMC11824804 22198637

[34] McCann JV Xiao L Kim DJ Khan OF Kowalski PS Anderson DG. Endothelial miR-30c suppresses tumor growth via inhibition of TGF-beta-induced Serpine1. J Clin Invest (2019) 129(4):1654–70. 10.1172/JCI123106 PMC6436861 30855280

[35] Calvisi DF Ladu S Gorden A Farina M Conner EA Lee JS. Ubiquitous activation of Ras and Jak/Stat pathways in human HCC. Gastroenterology (2006) 130(4):1117–28. 10.1053/j.gastro.2006.01.006 16618406

[36] Delire B Starkel P. The Ras/MAPK pathway and hepatocarcinoma: pathogenesis and therapeutic implications. Eur J Clin Invest (2015) 45(6):609–23. 10.1111/eci.12441 25832714

[37] Guan YS La Z Yang L He Q Li P. p53 gene in treatment of hepatic carcinoma: status quo. World J Gastroenterol (2007) 13(7):985–92. 10.3748/wjg.v13.i7.985 PMC4146884 17373730

[38] Morell CM Fiorotto R Fabris L Strazzabosco M. Notch signalling beyond liver development: emerging concepts in liver repair and oncogenesis. Clin Res Hepatol Gastroenterol (2013) 37(5):447–54. 10.1016/j.clinre.2013.05.008 23806629

[39] Zhou SL Zhou ZJ Hu ZQ Huang XW Wang Z Chen EB. Tumor-Associated Neutrophils Recruit Macrophages and T-Regulatory Cells to Promote Progression of Hepatocellular Carcinoma and Resistance to Sorafenib. Gastroenterology (2016) 150(7):1646–58.e17. 10.1053/j.gastro.2016.02.040 26924089

[40] Zhang Q He Y Luo N Patel SJ Han Y Gao R. Landscape and Dynamics of Single Immune Cells in Hepatocellular Carcinoma. Cell (2019) 179(4):829–45.e20. 10.1016/j.cell.2019.10.003 31675496

[41] Fu J Xu D Liu Z Shi M Zhao P Fu B. Increased regulatory T cells correlate with CD8 T-cell impairment and poor survival in hepatocellular carcinoma patients. Gastroenterology (2007) 132(7):2328–39. 10.1053/j.gastro.2007.03.102 17570208

[42] Chinai JM Janakiram M Chen F Chen W Kaplan M Zang X. New immunotherapies targeting the PD-1 pathway. Trends Pharmacol Sci (2015) 36(9):587–95. 10.1016/j.tips.2015.06.005 PMC4562806 26162965

[43] Giannini E Aglitti A Borzio M Gambato M Guarino M Iavarone M. Overview of Immune Checkpoint Inhibitors Therapy for Hepatocellular Carcinoma, and The ITA.LI.CA Cohort Derived Estimate of Amenability Rate to Immune Checkpoint Inhibitors in Clinical Practice. Cancers (2019) 11(11). 10.3390/cancers11111689 PMC6896125 31671581

[44] Street SE Trapani JA MacGregor D Smyth MJ. Suppression of lymphoma and epithelial malignancies effected by interferon gamma. J Exp Med (2002) 196(1):129–34. 10.1084/jem.20020063 PMC2194011 12093877

[45] Street SE Cretney E Smyth MJ. Perforin and interferon-gamma activities independently control tumor initiation, growth, and metastasis. Blood (2001) 97(1):192–7. 10.1182/blood.v97.1.192 11133760

[46] Shankaran V Ikeda H Bruce AT White JM Swanson PE Old LJ. IFNgamma and lymphocytes prevent primary tumour development and shape tumour immunogenicity. Nature (2001) 410(6832):1107–11. 10.1038/35074122 11323675

[47] Mitra-Kaushik S Harding J Hess J Schreiber R Ratner L. Enhanced tumorigenesis in HTLV-1 tax-transgenic mice deficient in interferon-gamma. Blood (2004) 104(10):3305–11. 10.1182/blood-2004-01-0266 15292059

[48] Kaplan DH Shankaran V Dighe AS Stockert E Aguet M Old LJ. Demonstration of an interferon gamma-dependent tumor surveillance system in immunocompetent mice. Proc Natl Acad Sci U S A (1998) 95(13):7556–61. 10.1073/pnas.95.13.7556 PMC22681 9636188

[49] Wang L Wang Y Song Z Chu J Qu X. Deficiency of interferon-gamma or its receptor promotes colorectal cancer development. J Interferon Cytokine Res (2015) 35(4):273–80. 10.1089/jir.2014.0132 25383957

[50] Bao S Wu Q McLendon RE Hao Y Shi Q Hjelmeland AB. Glioma stem cells promote radioresistance by preferential activation of the DNA damage response. Nature (2006) 444(7120):756–60. 10.1038/nature05236 17051156

[51] Huang Z Cheng L Guryanova OA Wu Q Bao S. Cancer stem cells in glioblastoma–molecular signaling and therapeutic targeting. Protein Cell (2010) 1(7):638–55. 10.1007/s13238-010-0078-y PMC4875273 21203936

[52] Schonberg DL Lubelski D Miller TE Rich JN. Brain tumor stem cells: Molecular characteristics and their impact on therapy. Mol Aspects Med (2014) 39:82–101. 10.1016/j.mam.2013.06.004 23831316 PMC3866208

[53] Chen J Ji T Wu D Jiang S Zhao J Lin H. Human mesenchymal stem cells promote tumor growth via MAPK pathway and metastasis by epithelial mesenchymal transition and integrin alpha5 in hepatocellular carcinoma. Cell Death Dis (2019) 10(6):425. 10.1038/s41419-019-1622-1 31142737 PMC6541606

[54] Kuninty PR Bansal R De Geus SWL Mardhian DF Schnittert J van Baarlen J. ITGA5 inhibition in pancreatic stellate cells attenuates desmoplasia and potentiates efficacy of chemotherapy in pancreatic cancer. Sci Adv (2019) 5(9):eaax2770. 10.1126/sciadv.aax2770 31517053 PMC6726450

[55] Lu L Xie R Wei R Cai C Bi D Yin D. Integrin alpha5 subunit is required for the tumor supportive role of fibroblasts in colorectal adenocarcinoma and serves as a potential stroma prognostic marker. Mol Oncol (2019) 13(12):2697–714. 10.1002/1878-0261.12583 PMC6887586 31600854

[56] Sodani K Patel A Kathawala RJ Chen ZS. Multidrug resistance associated proteins in multidrug resistance. Chin J Cancer (2012) 31(2):58–72. 10.5732/cjc.011.10329 22098952 PMC3777468

