# Supplementary figures and images for: An Inflammatory Response-Related Gene Signature Can Impact the Immune Status and Predict the Prognosis of Hepatocellular Carcinoma
Source: Front Oncol. 2021 Mar 22;11:644416. doi: 10.3389/fonc.2021.644416 (PMC8019928; doi:10.3389/fonc.2021.644416)

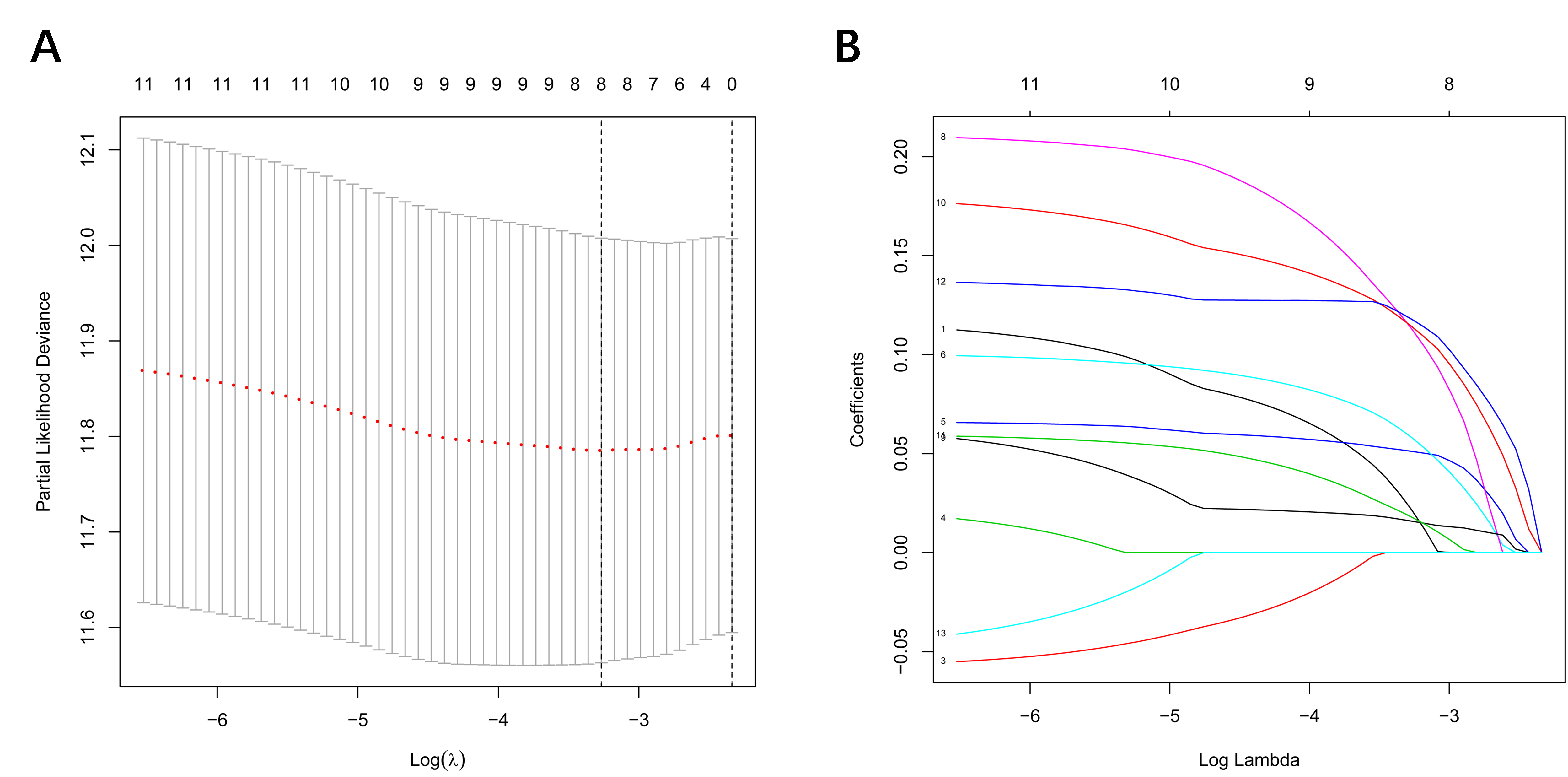

Supplement: Supplementary Figure 1 — Constructed an 8-gene signature in the TCGA cohort. (A) LASSO coefficient expression profiles of 13 candidate genes. (B) The penalty parameter (λ) in the LASSO model was selected through ten cross-validation. [file Image_1.tif]

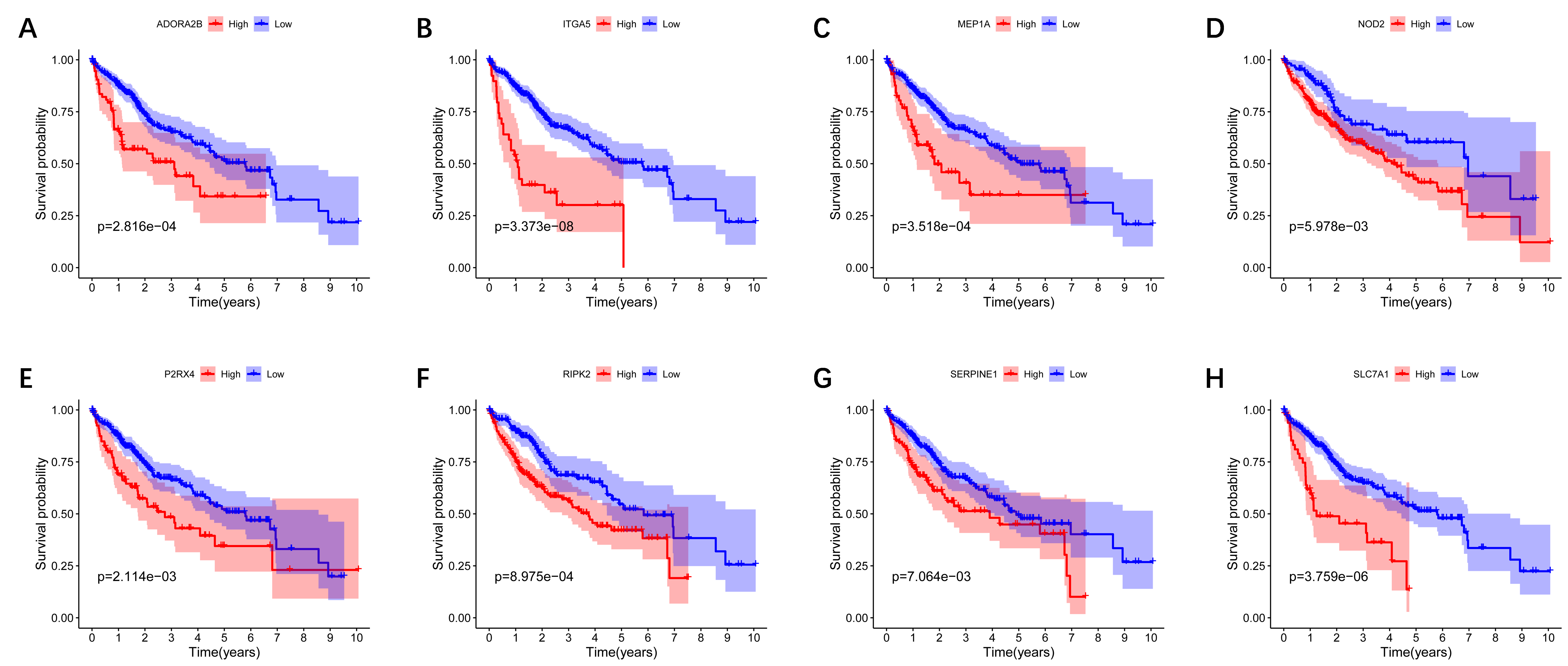

Supplement: Supplementary Figure 2 — Survival analysis of prognostic genes according to the optimal cut-off expression value. TCGA cohort (A–H). All adjusted P < 0.05. [file Image_2.tif]

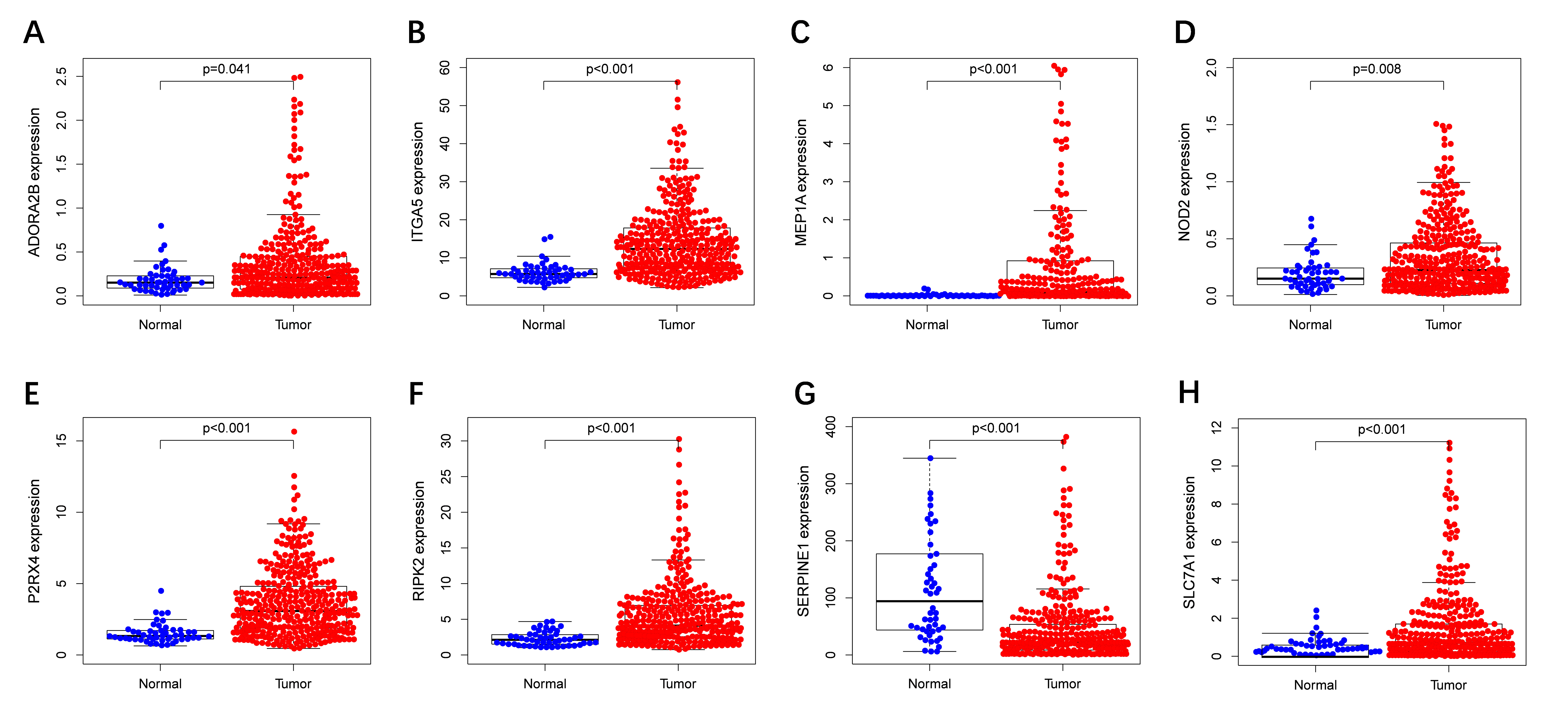

Supplement: Supplementary Figure 3 — Each prognostic gene expression between HCC tissues and adjacent non-tumorous tissues in TCGA. [file Image_3.tif]

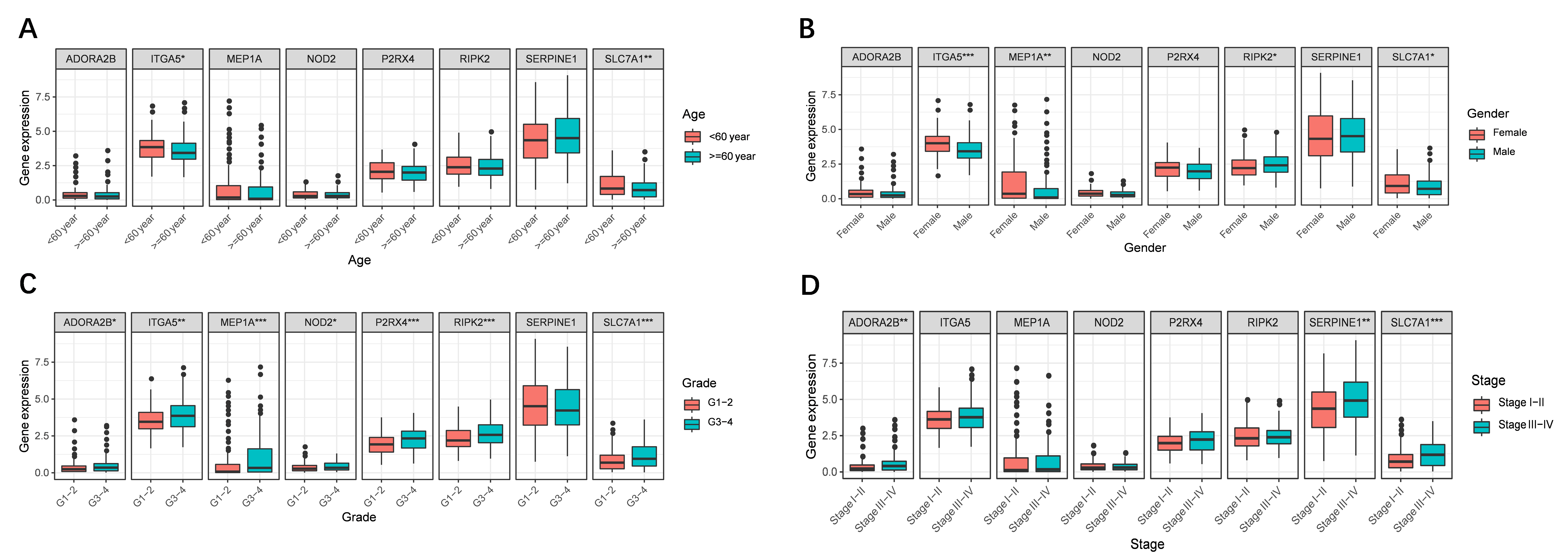

Supplement: Supplementary Figure 4 — Each prognostic gene expression in different groups divided by clinical characteristics. (A) Age. (B) Gender. (C) Tumor grade. (D) Tumor stage. [file Image_4.tif]

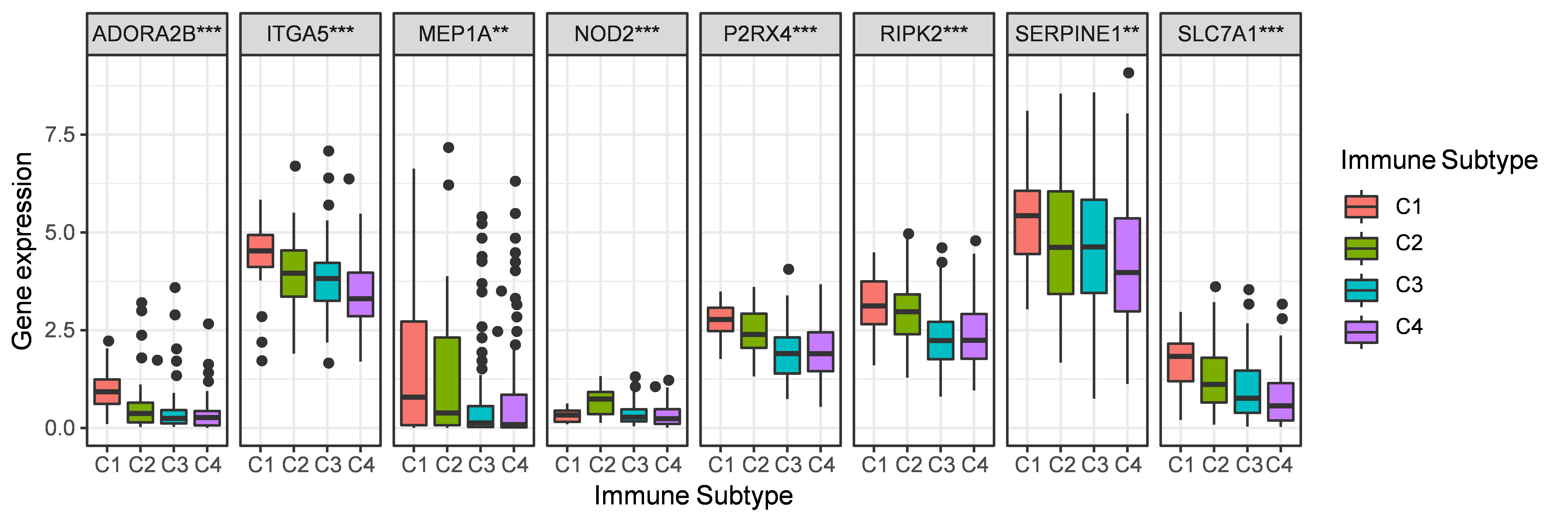

Supplement: Supplementary Figure 5 — The expression of prognostic genes in different immune infiltrate subtypes. [file Image_5.tif]

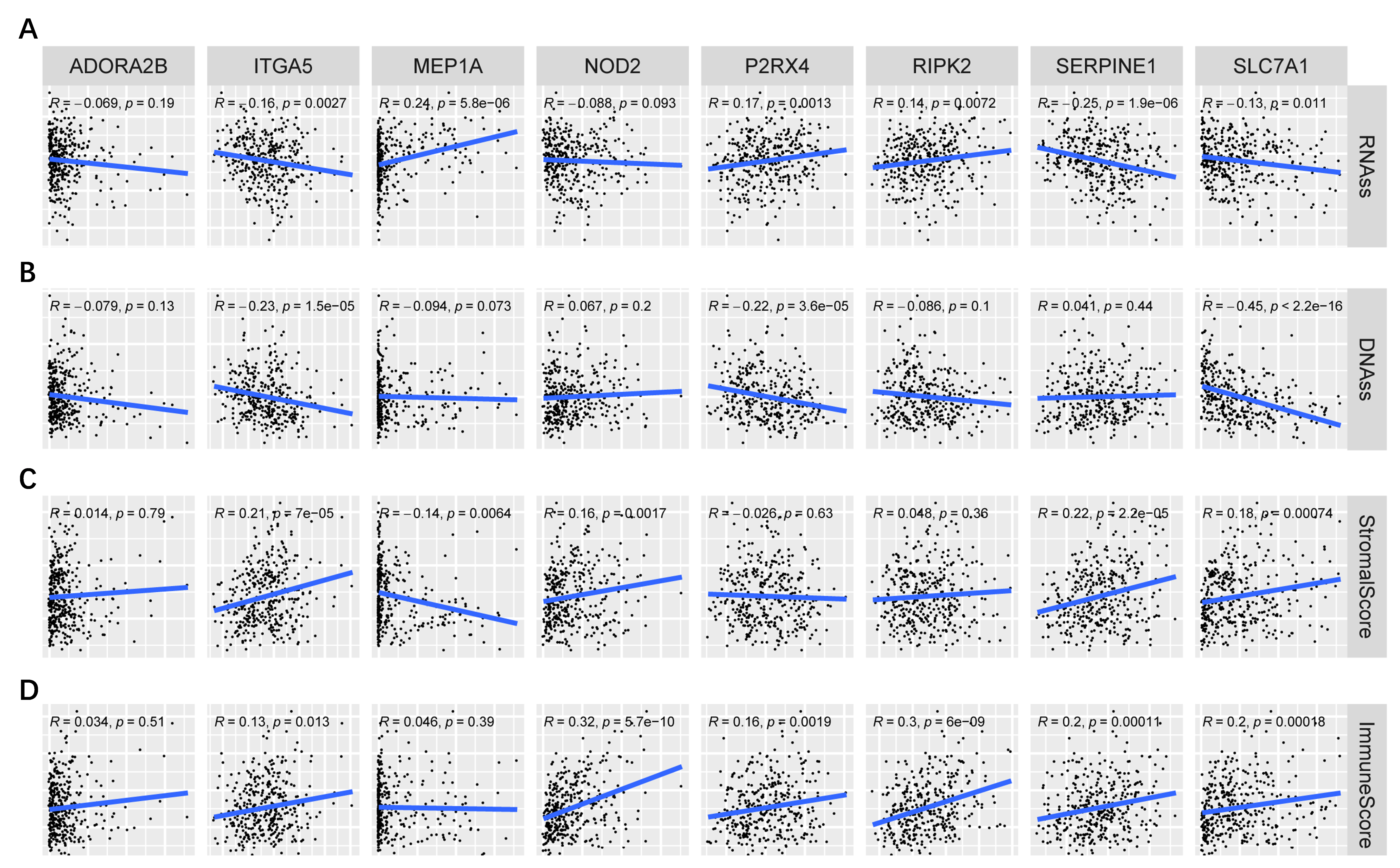

Supplement: Supplementary Figure 6 — The relationship of prognostic gene expression with RNAss, DNAss, Stromal Score and Immune Score. [file Image_6.tif]

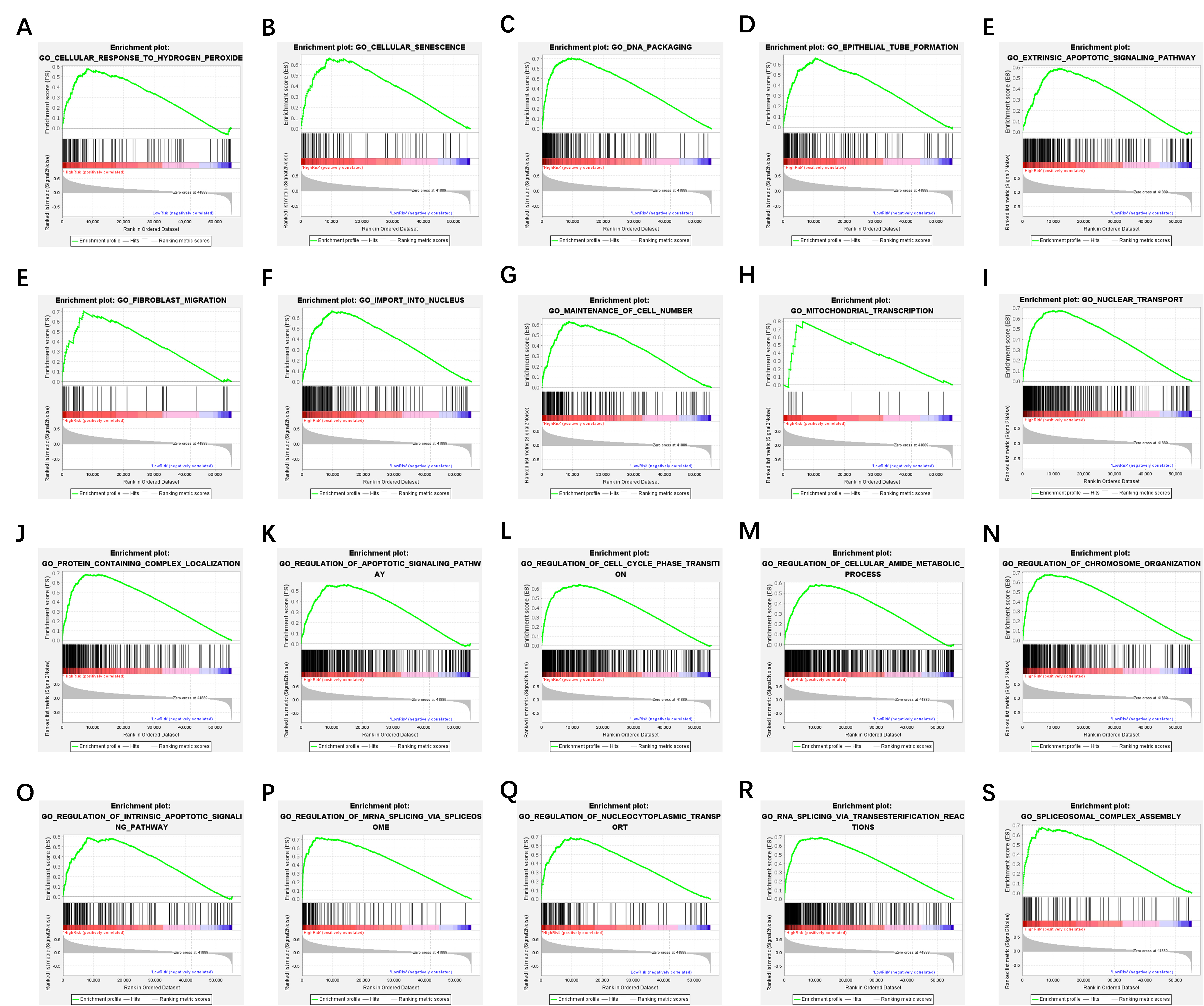

Supplement: Supplementary Figure 7 — Significant GO terms associated with high-risk score group. [file Image_7.tif]

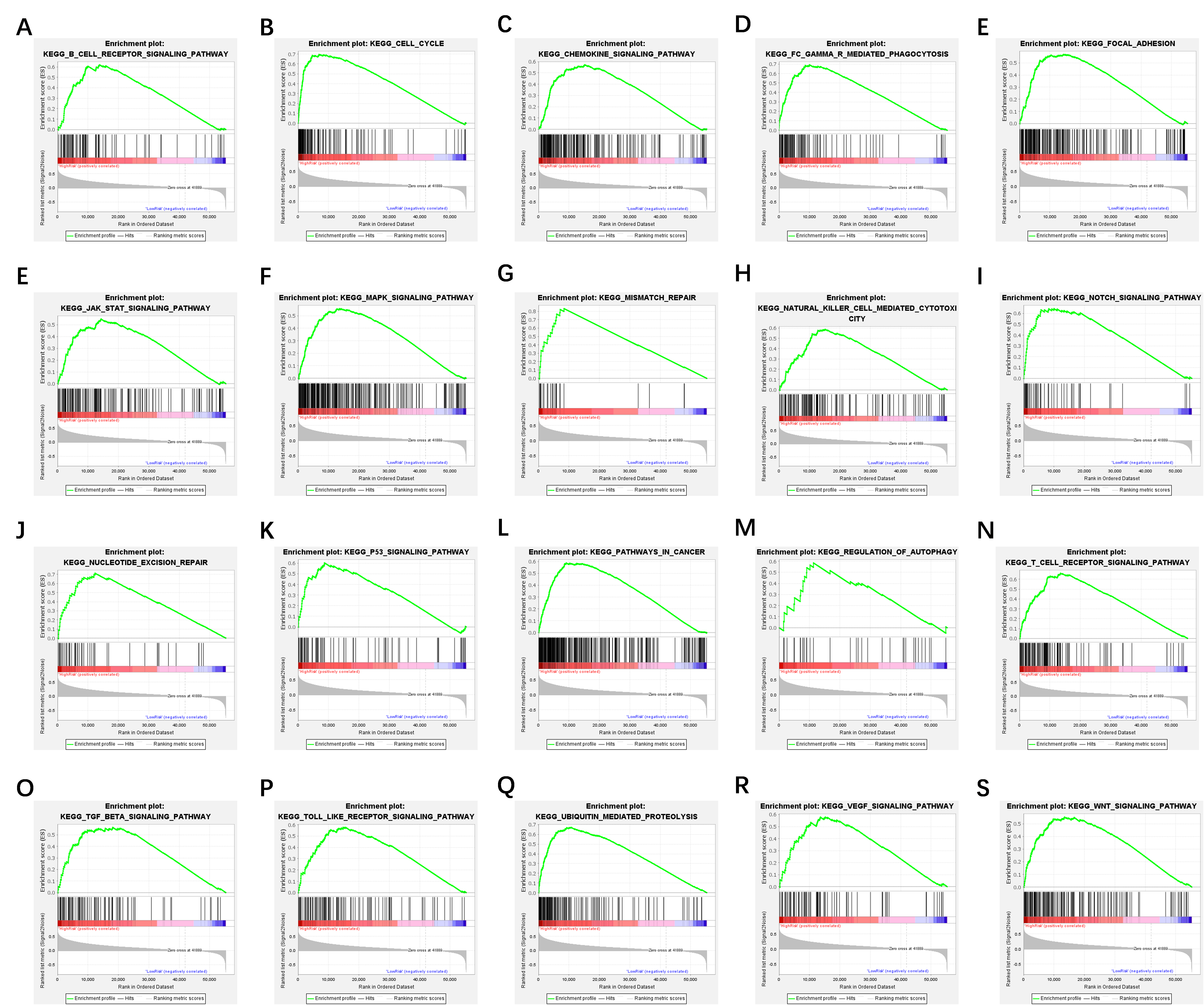

Supplement: Supplementary Figure 8 — Significant KEGG pathways associated with high-risk score group. [file Image_8.tif]

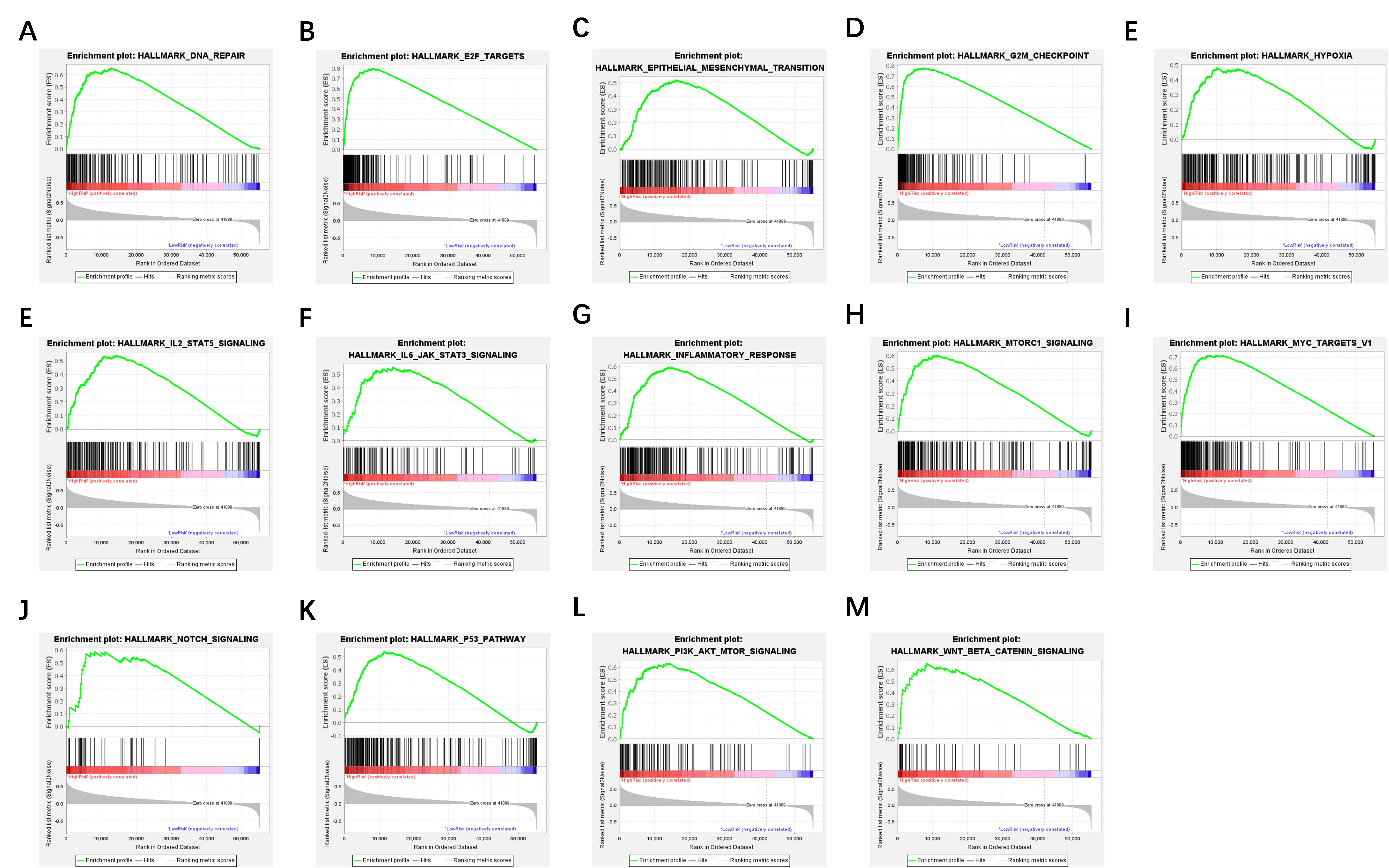

Supplement: Supplementary Figure 9 — Significant Hallmarks gene sets associated with high-risk score group. [file Image_9.tif]
